# Time-Course Evaluation of the In Vivo Resorption Process of Calcium Phosphates/Poly(lactide-*co*-glycolide) Composites Using Radiological Imaging and Histology

**DOI:** 10.3390/ijms27062549

**Published:** 2026-03-10

**Authors:** Shunsaku Takeishi, Kazuhiro Yasukawa, Maki Hiroshima, Chie Suzuki, Yasuhiro Magata

**Affiliations:** 1 Research and Development Department, Teijin Medical Technologies Co., Ltd., Okayama 7011221, Japan; shunt6545@yahoo.co.jp (S.T.); k.yasukawa@teijin.co.jp (K.Y.); 2 In Vivo Imaging Office, Division of Preeminent Bioimaging Research, Institute of Photonics Medicine, Hamamatsu University School of Medicine, Hamamatsu 4313192, Japan; 3 Molecular Imaging Laboratory, Division of Preeminent Bioimaging Research, Institute of Photonics Medicine, Hamamatsu University School of Medicine, Hamamatsu 4313192, Japan

**Keywords:** biomaterials, calcium phosphates, biodegradable polymers, composite materials, macrophages, cellular and tissue response, PET/CT, in vivo

## Abstract

There has been much development of composites of calcium phosphate and polymers for use as artificial bone, with other applications still ongoing, and clarification of the in vivo absorption mechanism is considered an important perspective. In order to clarify the absorption mechanism of bioabsorbable materials used for artificial bones and bone grafts, we prepared composites of calcium phosphate and polymers and conducted in vivo experiments in experimental animals using composites as implantation samples. Two typical types of calcium phosphate, β-tricalcium phosphate (β-TCP) and unsintered hydroxyapatite (uHA), were used as calcium phosphate, and copolymers of poly-dl-lactide-*co*-glycolide (PDLGA) and poly-l-lactide-*co*-glycolide (PLGA) were used as polymers. For samples composed of PDLGA and calcium phosphates, the weight ratios of calcium phosphate were set at 40% and 10% for uHA and 40% for β-TCP (uHA(40), uHA(10) and β-TCP(40), respectively). A composite sample of PLGA and uHA was also prepared with a weight ratio of 10% uHA (uHA(10)/PLGA), intending slow degradation of the polymer matrix compared to PDLGA. The samples were implanted in the metaphysis and diaphysis region of rabbits’ femur for up to 48 weeks. In this study, positron emission tomography/X-ray computed tomography (PET/CT) was used to continuously evaluate the changes in the samples and the accumulation of cells in the animals, and histological evaluation was performed, focusing on the time of characteristic changes in the PET/CT to confirm the cell types. The results are summarized as follows: (1) the absorption mechanism of the materials used in this study was suggested to be mainly phagocytosis by macrophages; (2) the disappearance rate was faster for β-TCP(40) compared with uHA(40); and (3) uHA(10), having a lower proportion of uHA, is not prone to aggregation and exhibited a similar disappearance result to β-TCP(40). These results suggest that phagocytosis by macrophages is the dominant path in resorption of the bioresorbable materials, and the resorption period varies depending on the type of polymer. It is important to optimize the type and amount of polymers and calcium phosphate in order to achieve a degradation rate of bioresorbable materials that corresponds to the extent of damage in the healing area.

## 1. Introduction

There exists a variety of bioresorbable polymers, but only a limited number of polymers have been commonly used in biomedical applications because it is necessary for the material to strictly fulfill the combination of requirements regarding safety, biocompatibility, physical and chemical properties, and biodegradability, as well as availability. Among such polymers, aliphatic polyesters of α-hydroxy acids, exemplified as polylactide (PLA) and polyglycolide (PGA), are widely utilized as bioresorbable implantable medical devices, including surgical sutures, membranes for tissue regeneration, and bone fixation devices.

Traditional bone fixation devices are made of metals, and accordingly, bioresorbable bone fixation devices have limitations in their clinical application due to their inferior mechanical properties compared to metallic materials. However, there are various advantages of biodegradable bone fixation devices over metallic ones. Unnecessity for a removal operation might be cited as a straightforward benefit. Another benefit is that prolonged interference with tendons and nerves [1] is avoided. It is also noted that the risk of implant-related stress-shielding [2] and infections are reduced with the use of biodegradable bone fixation devices.

The development of bioresorbable bone fixation devices using PLA, PGA, and their copolymers was begun in the late 1970s and early 1980s [3,4]. There were some challenges associated with the use of these polymers in bone fixation devices due to the fast degradation rate of PGA [5] and inferior mechanical strength of the materials compared to metallic implants [6]. As to the former issue, relatively slow-degrading PLA was extensively investigated as well as its copolymer with PGA [7]. Developments have been made on the materials suitable for targeted application by varying formulation, monomer ratio (for copolymers), fabrication method, etc. For the latter issue concerning mechanical properties, it was solved by toughening the material through the orientation of crystalline regions in polymers using various methods such as self-reinforcement [8,9], moderate uniaxial drawing to prevent fibrillation [10], and stereocomplex formation between enantiomeric PLA [11,12].

As mentioned above, bone fixation devices solely made of bioresorbable polymers have made considerable progress in terms of strength and/or degradation rate and have been put into practical use [13]. However, there was a need to develop a material with a set of solutions for the remaining problems of insufficient stiffness, the too-slow degradation rate of high-strength PLA, and lack of bioactivity [14]. Development of composite materials with calcium phosphate and bioglass is one of the solutions. Incorporation of these kinds of inorganic fillers brings hydrophilicity and bioactive properties and improves the modulus of the material. In addition, the basic degradation products of calcium phosphates could act as a buffer for the acidic degradation by-products of the aliphatic polyester, potentially preventing the creation of an unfavorable cellular environment caused by a decrease in pH [15,16].

Nowadays, many kinds of bioresorbable implants are commercially available with a variety of materials, applications, and characteristics [17,18]. The mechanism of the absorption of the materials and their degradation products in the body is thought to be dissolution in body fluids and phagocytosis by macrophages [19,20,21,22]. However, concerning composite materials with calcium phosphate, although there have been several reports of long-term implantation in bone and pathological evaluations [23,24,25], there are few cases where the mechanism has been thoroughly examined. Among these composite materials, as mentioned above, the disappearance mechanism of the polymer portion is well understood. On the other hand, the calcium phosphate component is believed to disappear through simple dissolution, phase transformation, fragmentation due to phagocytic action, and absorption through bone cells [26,27,28,29,30,31,32,33,34]. However, there are few detailed studies on the disappearance mechanism of materials that combine these calcium phosphates with lactic acid-based polymers.

In experiments involving the implantation of artificial materials into animals for evaluation, it is common practice to perform pathological evaluations of the implantation site after euthanasia. As part of the follow-up observations, X-ray computed tomography (CT) scans and similar procedures are sometimes conducted. However, the information obtained primarily pertains to the morphology of the surrounding hard tissues and the implanted material. Understanding how the changes observed during the period of animal care relate to the results of histological examinations can be challenging. In this regard, the combination of positron emission tomography (PET) and CT, referred to as PET/CT, provides functional information about tissues in addition to morphological data obtained through CT [35,36,37,38]. This is achieved by tracking the accumulation of compounds containing radioactive isotopes, often referred to as probes. By comparing the information obtained from pathological evaluations with the observations made through PET/CT, it is possible to capture transient conditions and temporal changes in areas of interest that may correlate with the results of the pathological assessments.

With this idea in mind, we implanted composite materials consisting of unsintered hydroxyapatite (uHA) [39,40,41,42,43] or β-tricalcium phosphate (β-TCP) [44,45], both of which had comparable particle sizes, with PLGA into the diaphysis and metaphysis of rabbit femurs. Subsequently, we conducted a detailed evaluation of the resorption behavior of these implants through longitudinal observations using PET/CT and pathological assessments after euthanasia.

## 2. Results

### 2.1. Rabbit Condition Before and Post-Implantation Surgery and Health Status During the Care Period

#### 2.1.1. Implantation Surgery

**Figure 1:**
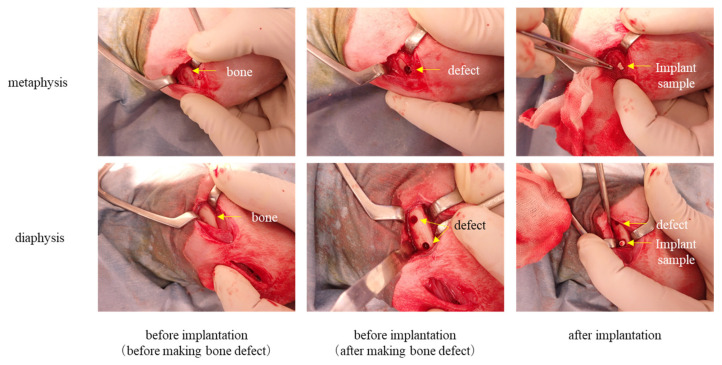
Implantation surgery. The skin was incised, the bone was exposed by dissection or an incision from the fascia area, and a non-penetrating hole was created in one location almost at the center of the metaphyseal end, gradually widening the diameter with an electric drill to finally create a hole of about ϕ4 × 7 (mm) in diameter to the inner surface of the cortical bone on the opposite side. Similarly, two ϕ3 × 5 (mm) defects were created in the metaphysis, one on the left and one on the right. Both defect sizes were smaller than the critical defect size. The samples were implanted in the bone defects and sutured to the muscle layer, subcutaneous tissue, and skin.

A representative example of the conditions during the implantation surgery is shown in Figure 1. The surgery followed the well-established procedure as described in the experimental methods. The procedure proceeded smoothly without any complications, and the rabbits awakened normally.

#### 2.1.2. Health Status During the Care Period

Throughout the care period, health monitoring was conducted by measuring body weight and collecting blood samples on a regular basis.

**Figure 2:**
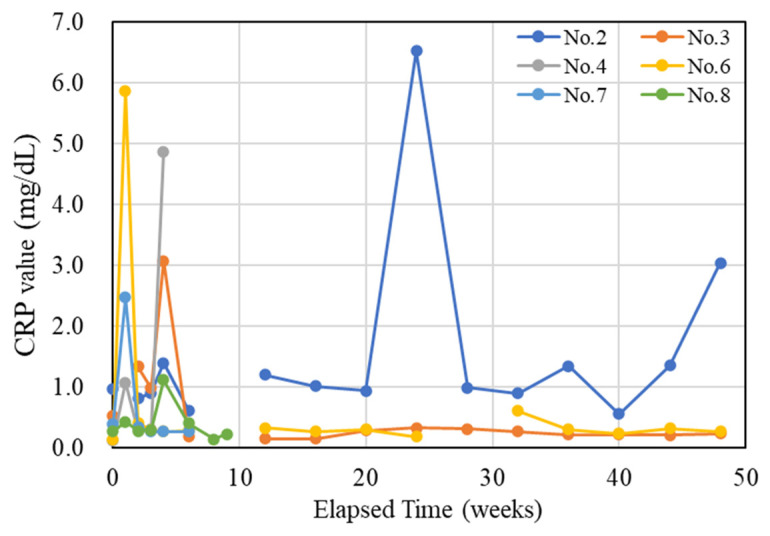
Chronological changes in CRP. Blood samples were collected once a month, and CRP was measured by ELISA. Serum from rabbits to be measured was divided into two tubes for CRP measurement, and the average value was used for evaluation. After collecting 0.5 mL of blood and allowing it to stand at room temperature for about 30 min, the serum was centrifuged (1400 G at 20 °C for 10 min), frozen, and measured at a later date.

Regarding body weight changes, while there were some fluctuations observed in all rabbits, they all progressed smoothly. The inflammatory status of the individuals during the care period was evaluated using C-reactive protein (CRP) tests. The results are presented in Figure 2. Quantitatively, there were individual differences, but as a general trend, they were similar. At 1 week post-implantation surgery, there was a strong inflammatory response, which was expected as this was still a period where the effects of the surgery were evident, resulting in elevated values. Subsequently, after the inflammation level decreased and stabilized, there was a resurgence of inflammation at 4 weeks post-implantation surgery. The 4-week post-implantation surgery period roughly coincided with the increase to a peak in 2-[^18^F]-Fluoro-2-deoxy-d-glucose (FDG) levels, as will be discussed later. Afterward, the inflammation levels decreased and stabilized again. Only Rabbit 2 consistently displayed higher inflammation levels during the stabilization phase compared with the other rabbits, and it exhibited a sudden increase at 24 weeks post-implantation surgery and displayed an upward trend from 36 to 48 weeks post-implantation surgery.

### 2.2. Changes in Appearance of CT Volume Rendering Images of Implantation Materials

**Figure 3:**
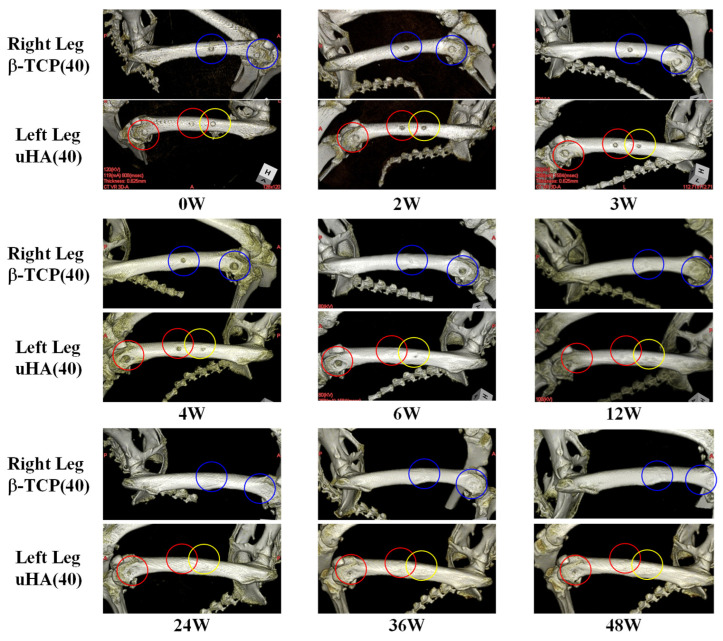
Changes in appearance of CT volume rendering images of materials implanted in Rabbit No. 3. Circles indicate the sites where the materials were implanted (blue: β-TCP(40), red: uHA(40), yellow: none (defect)).

The results from volume rendering of femoral CT scans are presented. Here, we show the results of observations conducted over 48 weeks in Rabbit No. 3 (Figure 3) and Rabbit No. 6 (Supplementary Figure S1) as representative cases. In the volume rendering images, the following trends were observed. At the metaphysis, there was no apparent difference between uHA(40) and β-TCP(40), and throughout the observation period, implant material remnants and traces suggestive of the implantation were observed. On the other hand, in the diaphysis, at 48 weeks post-implantation surgery, no residual was observed for β-TCP(40), while implant material remnants and traces suggestive of the implantation were observed for uHA(40). In the case of Rabbit No. 6, observations at the metaphysis showed that, for both uHA(10) and uHA(10)/PLGA, implant material remnants and traces suggestive of the implantation were observed even at 36 weeks post-implantation surgery. However, in the diaphysis, uHA(10) showed no implant material remnants and nothing looking like traces of the implantation at around 24 weeks post-implantation surgery, whereas uHA(10)/PLGA continued to display such structures even at 36 weeks post-implantation surgery.

From the results obtained, it can be inferred that β-TCP(40) and uHA(10) have largely disappeared at least by 48 weeks post-implantation surgery. In contrast, uHA(40) retains some form of trace or remnant that may be associated with the implant or its degradation products. Furthermore, uHA(10)/PLGA clearly suggests the persistence of uHA material.

### 2.3. Quantitative Evaluation of Implantation Materials—PET/CT

The temporal changes related to implantation materials and bone regeneration were evaluated using CT scans. Furthermore, the temporal changes in cellular gathering and dispersion primarily focused on macrophages were assessed using FDG, and the temporal changes in the cell histological aspects related to bone regeneration were evaluated using ^18^F-sodium fluoride (NaF).

#### 2.3.1. Femoral Diaphysis

**Figure 4:**
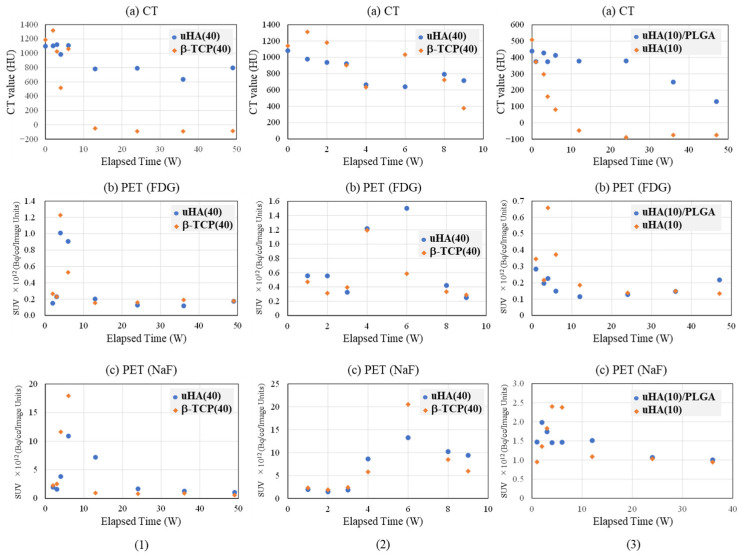
Chronological changes in CT value and PET signal of bone marrow region of implantation area in the diaphysis after implantation surgery. (**1**) shows chronological changes in Rabbit No. 3 (**a**) CT values, (**b**) FDG-Standardized uptake value (SUV) and (**c**) NaF-SUV. (**2**) shows chronological changes in Rabbit No. 8 (**a**) CT values, (**b**) FDG-SUV and (**c**) NaF-SUV. (**3**) shows chronological changes in Rabbit No. 6 (**a**) CT values, (**b**) FDG-SUV and (**c**) NaF-SUV.

**Figure 5:**
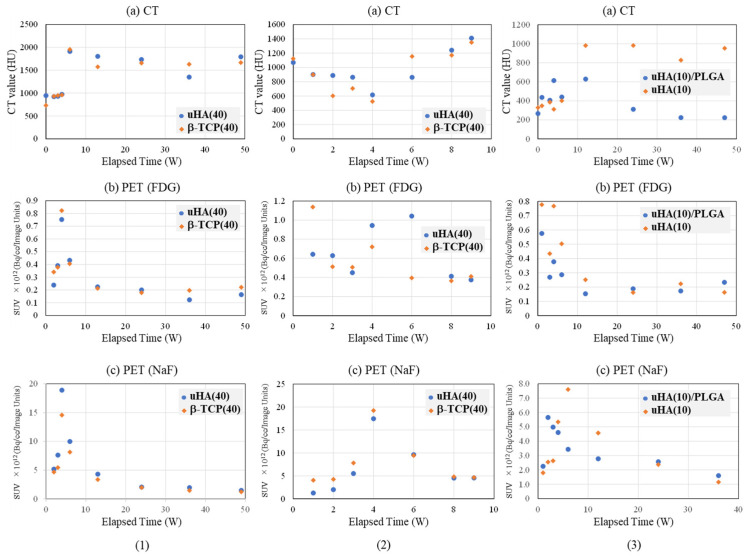
Chronological changes in CT value and PET signal of cortical bone region of implantation area in the diaphysis after implantation surgery. (**1**) shows chronological changes in Rabbit No. 3 (**a**) CT values, (**b**) FDG-SUV and (**c**) NaF-SUV. (**2**) shows chronological changes in Rabbit No. 8 (**a**) CT values, (**b**) FDG-SUV and (**c**) NaF-SUV. (**3**) shows chronological changes in Rabbit No. 6 (**a**) CT values, (**b**) FDG-SUV and (**c**) NaF-SUV.

Figure 4 and Figure 5 provide representative examples of CT, FDG, and NaF results in the femoral diaphysis (Figure 4(1) and Figure 5(1): Rabbit No. 3, Figure 4(2) and Figure 5(2): Rabbit No. 8, and Figure 4(3) and Figure 5(3): Rabbit No. 6).

In Figure 4, the results are shown for the bone marrow region within the implantation holes, while Figure 5 displays the results for the cortical bone region within the implantation holes. The trends in the temporal changes in the signal were often similar to those in the metaphysis. However, there was a tendency for the timing of the peaks to exhibit less variability compared with the metaphysis. This is thought to be due to the fact that the bone structure around the implantation materials is simpler than that in the metaphysis, that bone marrow regeneration is not as robust in these areas as in the metaphysis, and that there is less bone movement around implantation in the diaphysis than in the metaphysis. Here are some notable features in this area.

##### Bone Marrow Region

First, the temporal changes in CT are described. In all samples, significant values were observed at 0 weeks post-implantation surgery, attributable to the implantation itself. In the bone marrow region around the implantation site, both uHA(40) and β-TCP(40) showed little change until 3 weeks post-implantation surgery and subsequently declined towards 4 weeks post-implantation surgery. Subsequently, from 6 to 8 weeks post-implantation surgery, there was a temporary increase, followed by another decline, eventually reaching a plateau around 12 weeks post-implantation surgery. This trend was also observed in the bone marrow region at the metaphysis, but it was more pronounced in the diaphysis, with β-TCP(40) exhibiting larger changes. When reaching the stable level, the CT values for uHA(40) were greater than those for β-TCP(40), with the former being in the range of 600–800 HU and the latter approximately up to 100 HU. This suggests that, ultimately, β-TCP(40) had nearly disappeared, while uHA(40) remained, which is consistent with the findings from subsequent histological evaluations described later. In the case of Rabbit No. 8, the peak occurred at 8 weeks post-implantation surgery. However, the CT values at 6 weeks, 8 weeks, and 9 weeks post-implantation surgery were very close, suggesting that there was essentially no significant difference in trends among the specimens (although, it should be noted that only Rabbit No. 8 was observed at 8 weeks and 9 weeks post-implantation surgery). The uHA(10) sample implanted in Rabbit No. 6 had a lower uHA weight ratio of 10%, resulting in a smaller quantity of uHA compared with other specimens. Consequently, the initial CT value was lower than uHA(40) and β-TCP(40), similar to what was observed in the metaphysis. The CT values gradually decreased post-implantation surgery and did not show a peak. They reached a plateau around −100 HU from 12 to 24 weeks post-implantation surgery, similar to the behavior observed in β-TCP(40). For uHA(10)/PLGA, after an initial gradual increase, there was a gentle decline post-implantation surgery from around 24 weeks onwards, and even at 48 weeks post-implantation surgery, it was in a stage of gradual decline. This indicates that uHA(10) had largely disappeared, while uHA(10)/PLGA remained, consistent with the results of histological evaluations described later.

Next, the temporal changes in PET signals are described. Regarding FDG, when the ROI was set in the bone marrow region of the implantation holes, it exhibited a similar trend in all cases. In most specimens, it increased post-implantation surgery and reached its peak at around 4 weeks post-implantation surgery. For some specimens where the peak was observed at 6 weeks post-implantation surgery, the values were nearly unchanged compared with those at 4 weeks post-implantation surgery. Essentially, this suggests a similar state in these specimens. This is consistent with the observations in the metaphysis, where an increase in FDG was accompanied by a decrease in CT values, synchronizing with the disappearance of the implant. However, just in the case of uHA(10)/PLGA in Rabbit No. 6, while it also reached its peak at 4 weeks post-implantation surgery like the others, the increase was smaller. Throughout the observation period, it exhibited a state that was almost a monotonic decrease, and as mentioned earlier, there was no clear decrease in CT values. Regarding NaF, it peaked at 6 weeks post-implantation surgery for both uHA(40) and β-TCP(40). However, in both cases, there were instances where the decline in CT values temporarily stopped or even reversed when NaF peaked or a little later than the peak of NaF. In this respect, as mentioned above, the temporal change in NaF was synchronized with the temporal change in bone regeneration and was a good representation of the temporal change in bone regeneration. On the other hand, in the case of uHA(10), while there was an increase and peak in NaF, it was noticeably smaller when compared with uHA(40) and β-TCP(40). Additionally, there was no clear reduction in the rate of CT value decrease or temporary CT value increase as observed in uHA(40) and β-TCP(40).

##### Cortical Bone Region

First, the temporal changes in CT are described. Except for Rabbit No. 6, both uHA(40) and β-TCP(40) showed a general decrease or remained relatively stable towards 4 weeks post-implantation surgery. Subsequently, there was a significant increase around 6 weeks post-implantation surgery. Subsequently, there was a tendency for the CT values to maintain a nearly constant level. However, β-TCP(40) exhibited a slight decrease at around 12 weeks post-implantation surgery before stabilizing. During this time, the CT values for both were generally around 1500 HU. This reflects the initiation of cortical bone regeneration relatively early post-implantation surgery and suggests that implant disappearance and bone regeneration occur concurrently. By around 24 weeks post-implantation surgery, the implant had almost disappeared from the cortical bone region, indicating substantial cortical bone regeneration. This trend was also similar in Rabbit No. 6 with uHA(10), but the CT values showed a rapid increase at 12 weeks post-implantation surgery, which was notably slower than in the case of Rabbit No. 3. Additionally, the CT values at the plateau were around 1000 HU, which was lower compared with uHA(40) and β-TCP(40). This suggests that adequate bone regeneration might not have occurred at this point. On the other hand, uHA(10)/PLGA showed a small increase up to 12 weeks post-implantation surgery, followed by a gradual decrease, stabilizing at CT values equivalent to those at 0 weeks post-implantation surgery.

Next, the temporal changes in PET signals are described. Regarding FDG, the trends in the cortical bone region were similar to those in the bone marrow region. In other words, FDG levels increased post-implantation surgery and peaked at around 4 weeks post-implantation surgery. However, while in the bone marrow region, the FDG increase was accompanied by a decrease in CT values, in the cortical bone region, there was relatively little change. After FDG peaked and decreased (or concurrently with the decrease), CT values in the cortical bone region increased. On the other hand, with respect to NaF, in most specimens, there was an initial increase post-implantation surgery, and NaF levels peaked at around 4 weeks post-implantation surgery. However, in the case of uHA(10) in Rabbit No. 6, NaF peaked at 6 weeks post-implantation surgery, and similar to the result observed in the metaphysis, CT values started to rise after NaF increased. In contrast, for uHA(10)/PLGA in Rabbit No. 6, regardless of the ROI location, NaF levels peaked at 2 weeks post-implantation surgery and then monotonously decreased. These values were roughly similar to those seen in other specimens at 2 weeks post-implantation surgery. As described above, in the cortical bone region, similar to the bone marrow region, the changes in CT values and the behavior of FDG were found to be related, indicating their involvement in the removal of the implant. This result was similar to the result in the metaphysis. The behavior of NaF also synchronized with the changes in CT values (with NaF peaking slightly earlier or at the same time as the rapid increase in CT values), suggesting a correlation with bone regeneration. Moreover, these phenomena support the notion, as mentioned earlier, that “CT values decrease with the disappearance of implantation materials, and CT values increase with cortical bone regeneration simultaneously.”

#### 2.3.2. Femoral Bone Defect Area

**Figure 6:**
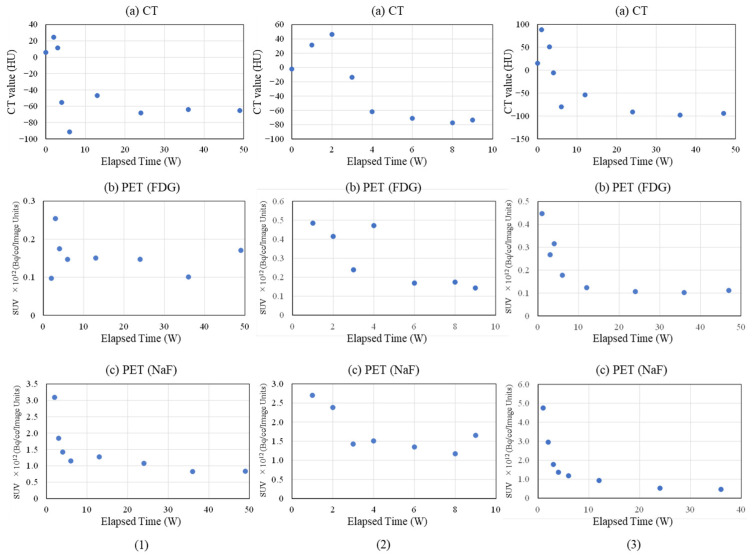
Chronological changes in CT value and PET signal of bone marrow region in the femoral bone defect after implantation surgery. (**1**) shows chronological changes in Rabbit No. 3 (**a**) CT values, (**b**) FDG-SUV and (**c**) NaF-SUV. (**2**) shows chronological changes in Rabbit No. 8 (**a**) CT values, (**b**) FDG-SUV and (**c**) NaF-SUV. (**3**) shows chronological changes in Rabbit No. 6 (**a**) CT values, (**b**) FDG-SUV and (**c**) NaF-SUV.

**Figure 7:**
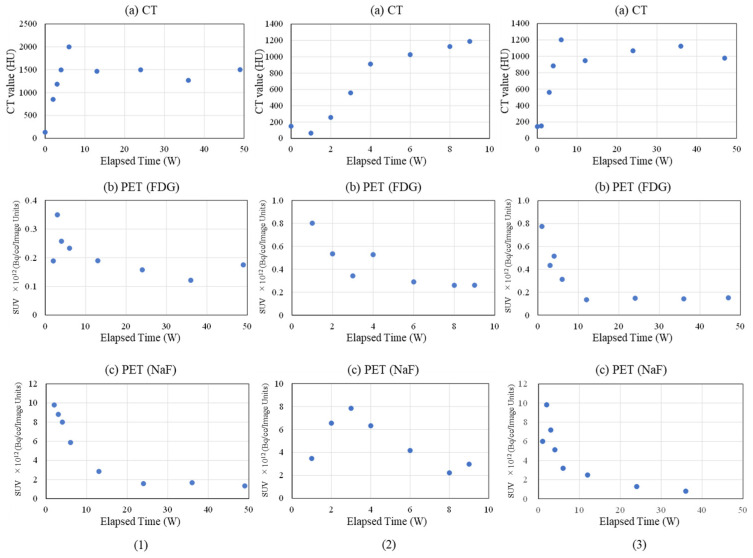
Chronological changes in CT value and PET signal of cortical bone region in the femoral bone defect after implantation surgery. (**1**) shows chronological changes in Rabbit No. 3 (**a**) CT values, (**b**) FDG-SUV and (**c**) NaF-SUV. (**2**) shows chronological changes in Rabbit No. 8 (**a**) CT values, (**b**) FDG-SUV and (**c**) NaF-SUV. (**3**) shows chronological changes in Rabbit No. 6 (**a**) CT values, (**b**) FDG-SUV and (**c**) NaF-SUV.

The representative results of CT, FDG, and NaF in PET/CT for the femoral bone defect area within the femoral diaphysis are shown in Figure 6 and Figure 7 (Figure 6(1) and Figure 7(1): Rabbit No. 3, Figure 6(2) and Figure 7(2): Rabbit No. 8, and Figure 6(3) and Figure 7(3): Rabbit No. 6). Figure 6 presents the results for the bone marrow region of the implantation hole, while Figure 7 shows the results for the cortical bone region of the implantation hole. Furthermore, a bone defect of the same size as the implant (ϕ3 × 5 mm) was created proximal to the site where the samples were implanted in the left femur, which is the side closer to the hip than the knee.

##### Bone Marrow Region

In the bone marrow region of the bone defect, the CT value was approximately 0 HU at the time of implantation (0 weeks post-implantation surgery). Subsequently, it increased and reached its peak at approximately 1–2 weeks post-implantation surgery, with CT values ranging from several tens to around 100 HU. Afterward, it decreased and stabilized at a constant value from 12 weeks post-implantation surgery onwards, with a final CT value ranging from −70 HU to −100 HU. In other words, this period represents the healing process when only a bone defect was created without the presence of an implant, indicating the course of soft tissue regeneration.

Next, the temporal changes in PET signals are described. First, FDG is described. FDG peaked at 3 to 4 weeks post-implantation surgery, and this peak occurred after the CT values had already reached their peak. As for NaF, it showed its maximum value at the beginning of the measurements, which was attributed to the implantation surgery. Furthermore, this maximum value was less than one-third to one-half of the peak value in the cortical bone region, which will be shown later.

##### Cortical Bone Region

In the cortical bone region of the implantation hole, the CT values were approximately 0 HU at post-implantation surgery week 0 and then increased and reached a peak around post-implantation surgery week 6, followed by a slight decrease until post-implantation surgery week 12, after which they exhibited a trend to stabilize at around 1500 HU. It is worth noting that only Rabbit No. 8 was observed at post-implantation surgery weeks 8 and 9. Rabbit No. 8 showed a continuous increase until post-implantation surgery week 9, but similar values were observed at weeks 6, 8, and 9 (only Rabbit No. 8 was observed at post-implantation surgery weeks 8 and 9). Rabbit No. 6 exhibited a similar trend, but the values after post-implantation surgery week 12 were slightly lower, with CT values around 1000–1200 HU. These results pertain to the cases where just bone defects were created, illustrating the course of cortical bone regeneration.

Next, the temporal changes in PET signals are described. Regarding FDG, similar to the bone marrow region, it peaked around post-implantation surgery week 3 or 4, but unlike the bone marrow region, it peaked before the CT values reached their peak. As for NaF, it peaked between post-implantation surgery weeks 1 and 3, also preceding the peak in CT values. In other words, similar to cases where implantation materials were placed, the maturation of regenerated bone occurred at the time of NaF peaking or slightly afterward.

#### 2.3.3. Implantation of Polymer Alone in the Femoral Diaphysis

**Figure 8:**
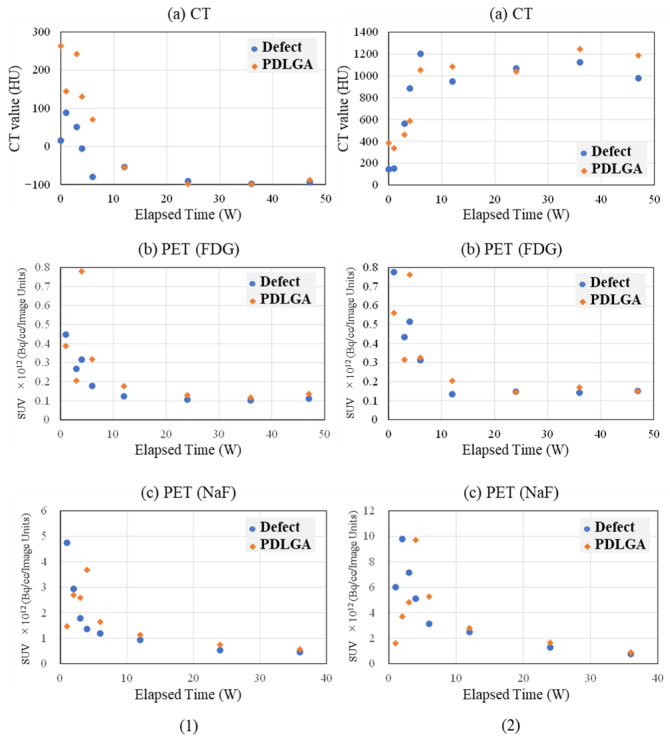
Chronological changes in CT value and PET signal of bone defect area and of implantation area in the diaphysis of Rabbit No. 6 after implantation surgery. The bone defects were created in the left and right femoral diaphysis of Rabbit No. 6, and the implant made entirely of the polymer PDLGA was implanted in the bone defect on the right side. (**1**) shows chronological changes in bone marrow region (**a**) CT values, (**b**) FDG-SUV and (**c**) NaF-SUV. (**2**) shows chronological changes in cortical bone region (**a**) CT values, (**b**) FDG-SUV and (**c**) NaF-SUV.

Lastly, we show the results of a specimen (Rabbit No. 6) where, in addition to the previously mentioned defect, a site proximal to the location where calcium phosphate was implanted in the right femoral bone was implanted with a polymer (PDLGA: the same polymer size as that used for implantation materials in the femur, measuring ϕ3 × 5 mm) that does not contain calcium phosphate. The results of CT values, FDG, and NaF when ROI size/position was limited to the implant’s bone marrow region are presented in Figure 8(1), while the results when limited to the cortical bone region are shown in Figure 8(2). For the sake of comparison, values for the bone defect area (as was shown in Figure 6(3) for the bone marrow region and Figure 7(3) for the cortical bone region) are overlaid in each set of results.

##### Bone Marrow Region

First, the changes in CT values are described. The CT values for PDLGA-only implantation, bone defect-only, uHA(10) implantation, and uHA(40) implantation were compared. Regarding the bone marrow region, when the polymer was implanted, the initial values were higher than in the case of the bone defect-only situation (Figure 6(1),(3) and Figure 8(1)). They decreased once and then increased around 3 weeks post-implantation surgery, after which they gradually decreased again, eventually reaching values that suggest the polymer had disappeared. The period required for the values to decrease to levels suggestive of disappearance was approximately 24 weeks post-implantation surgery, which was similar to the situation observed with uHA(10) (Figure 6(1),(3). The CT values for the bone defect-only case stabilized at approximately the same time. In the case of β-TCP(40), it showed a somewhat faster disappearance but followed a similar course as the other three cases. As previously mentioned, uHA(40) (Figure 4(1)) was the only one that exhibited a different trend, as it remained present even after 48 weeks post-implantation surgery. However, this was likely a unique sample within the scope of this study.

Next, the temporal changes in PET signals are described. In the region where the polymer was implanted, both FDG and NaF showed peaks at around 4 weeks post-implantation surgery, which were delayed compared with the CT value peaks. The timing of these peaks was similar to what was observed in the case of uHA(10).

##### Cortical Bone Region

First, the changes in CT values are described. In the cortical bone region, when only PDLGA was implanted, the increase in CT values was delayed compared with the increase observed in the case of bone defect-only samples. While the latter reached its maximum CT value at 4–6 weeks post-implantation surgery, the former reached its maximum value at 6 weeks post-implantation surgery and onwards. Subsequently, both exhibited similar changes (Figure 7(1),(3) and Figure 8(2)). On the other hand, when uHA(10) was implanted, the increase in CT values was notably delayed compared with the cases of bone defect-only samples and PDLGA-only implantation. The maximum CT value was reached at 12 weeks post-implantation surgery (Figure 5(3)). However, uHA(40), with a higher uHA content (similar changes were observed for β-TCP(40)), also showed changes similar to the bone defect-only cases (Figure 5(1) and Figure 7(1)). In other words, the time to reach the maximum value when the calcium phosphate content was high was shorter than that when the calcium phosphate content was low and was similar to that in the case of bone defect only. Moreover, the treatment involving bone defect only was also conducted in Rabbit No. 3 (Figure 7(1)), and the results from this case reproduced the previously mentioned results from Rabbit No. 6′s bone defect-only case (Figure 7(3) and Figure 8(2)). On the other hand, when uHA(40) or β-TCP(40) was implanted (Figure 5(1)), the period until the CT values reached their maximum was 6 weeks post-implantation surgery.

Next, the temporal changes in PET signals are described. When only PDLGA was implanted, FDG reached its peak earlier than the CT values reached a plateau. Both in the PDLGA implantation site and the bone-deficient site, the peaks occurred at approximately the same time. However, the peak value of FDG was higher in the PDLGA implantation site compared with the bone-deficient site. This is believed to be due to the removal of PDLGA. On the other hand, regarding NaF, both in the PDLGA implantation site and the bone-deficient site, CT values showed a rapid increase and reached a peak earlier compared with reaching a plateau (Figure 8(2)). However, the bone-deficient site reached its peak even earlier. This difference is believed to be related to the rate of bone regeneration (temporal changes in CT values). Based on these results, it can be inferred that NaF is involved in bone formation similarly to other sites, while FDG tends to show higher values when there are more substances that need to be eliminated.

### 2.4. Histological Evaluation

The histological evaluation is described below in chronological order of implantation periods. The implantation materials were placed in the same location for Rabbit No. 1, 2, 3, 4, 7, and 8, but only Rabbit No. 6 had different implants from other specimens (details are provided in Section 4). Rabbit No. 6 also underwent histological evaluation similar to the other samples.

#### 2.4.1. Evaluation of the Femoral Diaphysis

**Figure 9:**
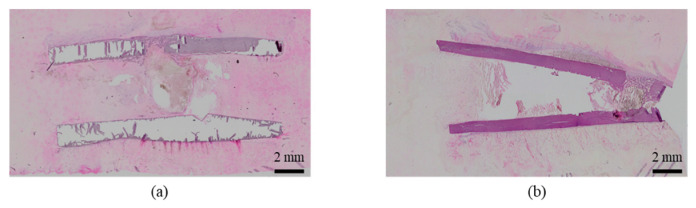
Representative histological images of the diaphysis in Rabbit-No. 1 at 1 week after implantation surgery. (**a**) HE image around the implant (uHA (40)) in the left diaphysis. (**b**) HE image around the implant (β-TCP (40)) in the left diaphysis.

The histological evaluation results of the femoral diaphysis 1 week post-implantation surgery are shown in Figure 9 (Supplementary Figure S11) (Rabbit No. 1). For both uHA(40) and β-TCP(40), regenerated bone was accompanied by osteoid, extended covering the outer side of the implantation hole, and formed a bridging structure, which, for uHA(40), covered approximately 1/3 to 1/2 of the thickness of the cortical bone and, for β-TCP(40), covered a little over 1/2 (Figure 9a,b), Supplementary Figure S11a–d). On the outer surface of the implantation hole, a slightly thicker fibrous tissue was observed. Bone marrow tissue was not observed between the regenerated bone, and there was no significant proliferation of granulation tissue. Furthermore, for uHA(40) and β-TCP(40), there was a slight infiltration of mononuclear cells observed within the regenerated bone.

**Figure 10:**
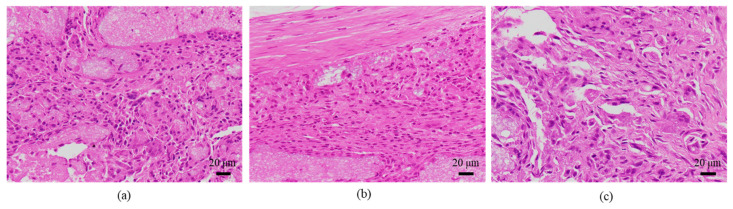
Representative histological images of the diaphysis in Rabbit-No. 4 at 4 weeks after implantation surgery (×400). (**a**) HE image of the implant (uHA (40)) in the left diaphysis. (**b**) HE image of the implant (uHA (40)) in the left diaphysis. (**c**) HE image of the implant (β-TCP (40)) in the left diaphysis.

The histological evaluation results of the diaphysis at 4 weeks post-implantation surgery are shown in Figure 10 (Supplementary Figure S12) (Rabbit No. 4). For uHA(40), implantation holes were observed in one side of the cortical bone, and the implantation material, separated from granulation tissue, extended slightly beyond the implantation hole onto the outer surface of the bone. Furthermore, the outer surface was covered by a thick fibrous tissue containing relatively dense collagenous fibers (stained red with EVG staining) (Supplementary Figure S12a,b). The absence of tissue transition between this surface fibrous tissue and the granulation tissue encapsulating the implantation material (Figure 10b, Supplementary Figure S12c) suggested that the surface fibrous tissue was derived from periosteal tissue. The granulation tissue near the implantation holes showed a slight increase in spindle-shaped fibroblast-like cells, but within the bone, it was primarily composed of macrophages including multinucleated giant cells, and fibroblasts were relatively scarce. These cells were intermingled with finely fragmented implantation material at the periphery of the implant, and clear formation of a cell layer was not observed in some areas, indicating that the encapsulation was somewhat incomplete (Figure 10a and Supplementary Figure S12d). From these series of phenomena, it is suggested that, at this stage, the activity of implantation material fragmentation and removal by inflammatory cells, primarily macrophages, was beginning to be actively carried out. Bone formation around the implantation material was relatively limited, being observed to a minor extent on the sides and in the deeper areas. In the right femoral diaphysis (β-TCP(40)), it was similar to the left side (uHA(40)), but there were some differences noted as mentioned below. The implantation hole on the right side was slightly narrower than on the left side, and the implantation materials that leaked outside the bone along with granulation tissue were fewer on the right side than on the left (Supplementary Figure S12e,f). The fibrous tissue covering its outer surface was thinner on the right side compared with the left side, and the collagenous fiber density on the right side was also lower (Supplementary Figure S12g). In the granulation tissue in which macrophages were the main group, there were slightly more fibroblasts and lymphocytes overall compared with the left side, and they encapsulated the implantation material almost entirely (Figure 10c, Supplementary Figure S12h). Bone formation around the implantation material was less pronounced on the right side compared with the left side, and it was observed only near the defect in the cortical bone.

**Figure 11:**
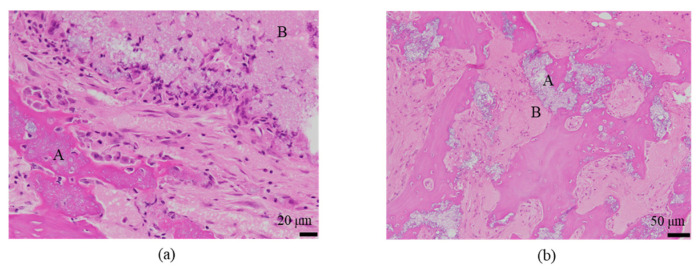
Representative histological images of the diaphysis in Rabbit-No. 7 at 6 weeks after implantation surgery. (**a**) HE image of the implant (uHA(40)) in the left diaphysis (×400). (**b**) HE image of the implant (β-TCP(40)) in the right diaphysis (×200). A, foreign materials observed inside the newly formed bone; B, foreign materials observed outside the newly formed bone.

The histological evaluation results of the diaphysis at 6 weeks post-implantation surgery are shown in Figure 11 (Supplementary Figure S13) (Rabbit No. 7). For uHA(40), a proliferation of newly formed bone containing the implant material and continuously continuing to one side of the cortical bone was observed within the bone marrow (Figure 11a,b). Similar to the results obtained from HE staining (Supplementary Figure S13c,d: A, B), EVG staining, and PAS staining at the metaphysis (Supplementary Figure S7e,f), foreign materials were observed both inside and outside the newly formed bone, and they were also present within the continuous cortical bone (Supplementary Figure S13c). Around the foreign materials outside the bone, there were macrophages, including a small amount of multinucleated giant cells, and fibroblasts, and white blood cells were also observed (Figure 11a, Supplementary Figure S13d). OC immunostaining showed a weak positive reaction on the foreign materials (Supplementary Figure S13g). As for β-TCP(40), similar to uHA(40), a proliferation of newly formed bone containing the implant material and continuously continuing to one side of the cortical bone was observed within the bone marrow (Supplementary Figure S13h,i). The foreign materials were observed both inside and outside the bone, and in some cases, they were even observed within the continuous cortical bone. However, the foreign materials outside the bone were somewhat less distinct compared with within the bone, likely due to cellular infiltration of macrophages and fibroblasts, and some bleeding (Supplementary Figure S13h,i). OC immunostaining was focally positive on the foreign materials outside the bone (Supplementary Figure S13m). From this series of phenomena, the reaction related to the removal of the implant was still actively ongoing, similar to what was observed at 4 weeks post-implantation surgery. Additionally, bone formation appeared to be more active compared with 4 weeks post-implantation surgery.

**Figure 12:**
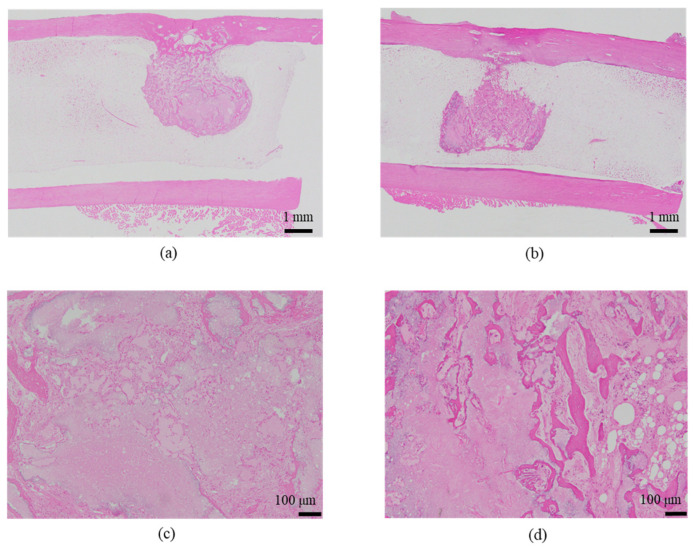
Representative histological images of the diaphysis in Rabbit-No. 8 at 9 weeks after implantation surgery. (**a**) HE image around the implant (uHA(40)) in the left diaphysis. (**b**) HE image around the implant (β-TCP(40)) in the right diaphysis. (**c**) HE image of the implant (uHA(40)) in the left diaphysis (×100). (**d**) HE image of the implant (β-TCP(40)) in the right diaphysis (×100).

The histological evaluation results of the diaphysis at 9 weeks post-implantation surgery are shown in Figure 12 (Supplementary Figure S14) (Rabbit No. 8). The condition of the femoral diaphysis on both sides was generally similar to that observed at 6 weeks post-implantation surgery (Rabbit No. 7), but the biological responses had progressed further than at 6 weeks. In the cortical bone region, osteoblasts and osteoclasts were observed in some areas on both sides, and the newly formed bone had fused, approaching the native thickness of the cortical bone, as compared with the femoral metaphysis (Figure 12a,b, Supplementary Figure S14a–d). Compared to 6 weeks post-implantation surgery (Rabbit No. 7), at 9 weeks post-implantation surgery (Rabbit No. 8), the newly formed bone had fused more extensively and appeared thicker. In the bone marrow region, on both sides of the femoral diaphysis, irregularly distributed newly formed bone containing foreign materials was observed. In areas with limited new bone formation, the presence of residual implantation materials was more pronounced, with a higher degree of infiltration by mainly macrophages (including giant cells) and fibroblasts compared with 6 weeks post-implantation surgery (Rabbit No. 7), while neutrophils were notably less abundant (Figure 12c,d, Supplementary Figure S14e,f). However, regarding the fragments of the implantation materials, uHA(40) had a larger number of larger fragments that remained.

**Figure 13:**
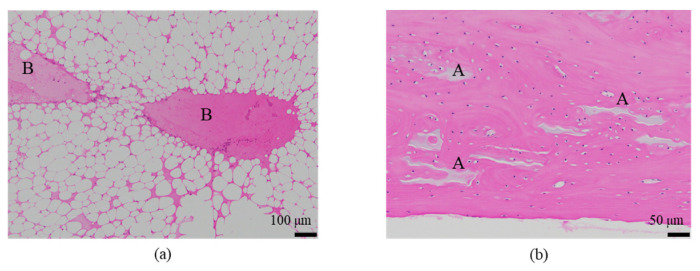
Representative histological images of the diaphysis in Rabbit-No. 3 at 48 weeks after implantation surgery. (**a**) HE image of the implant (uHA(40)) in the left diaphysis (×100). (**b**) HE image of the implant (β-TCP(40)) in the right diaphysis. A, foreign materials observed inside the newly formed bone; B, foreign materials observed outside the newly formed bone.

The histological evaluation results of the diaphysis at 48 weeks post-implantation surgery are shown in Figure 13 (Supplementary Figure S15) (Rabbit No. 3). On both the left and right sides, there were no apparent large masses of implantation materials within the cortical bone region and the bone marrow region that were presumed to be the area in which the implantation was buried (Supplementary Figure S15a,b,h,i). Within the bone marrow, there were some voids, areas of low cell density mainly consisting of adipocytes and areas of slightly higher cell density. Additionally, on the left side, foreign materials (B) were sporadically observed (Figure 13a, Supplementary Figure S15c). Furthermore, based on the state of cells primarily composed of macrophages including adipocytes, it was suggested that the elimination of implantation materials through phagocytosis had reached its final stages. For uHA(40), fragments of implantation materials including some larger ones were mainly distributed within the bone marrow (Figure 13a, Supplementary Figure S15c), which corroborated the results of the CT scans. Furthermore, internally, it appeared to contain plasma components including red and white blood cells, which showed a slightly intense red color by HE staining (Supplementary Figure S15g). These foreign bodies within the bone marrow showed staining characteristics similar to those at the left and right femoral metaphysis with EVG staining and PAS staining (Supplementary Figure S15d–f,j–l). The foreign bodies (A) within the bone were not as distinct as at the femoral metaphysis but were present in small amounts. In the right distal femoral diaphysis, they were observed to align within the cortical bone along the longitudinal axis of the bone. Positive reactions were observed with OC immunostaining, suggesting that bone regeneration was occurring (Figure 13b, Supplementary Figure S15m). Furthermore, similar to the results shown for the metaphysis, it was suggested that foreign bodies (A) were incorporated into the bone, while foreign materials (B) were subject to elimination.

**Figure 14:**
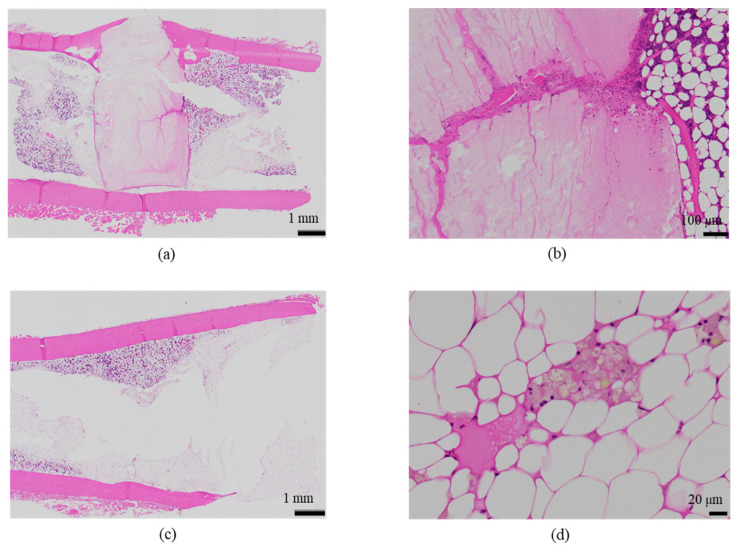
Representative histological images of the diaphysis in Rabbit-No. 6 at 48 weeks after implantation surgery. (**a**) HE image around the implant (uHA(10)/PLGA) in the left diaphysis. (**b**) PAS image around the implant (uHA(10)/PLGA) in the left diaphysis (×100). (**c**) HE image around the implant (uHA (10)) in the right diaphysis. (**d**) HE image of the implant (uHA (10)) in the right diaphysis (×400).

The histological evaluation results of the diaphysis in Rabbit No. 6 at 48 weeks post-implantation surgery are shown in Figure 14 (Supplementary Figure S16). For uHA(10)/PLGA, in the left distal femoral diaphysis, the implantation materials were observed to almost transversely span the femoral diaphysis from the implantation hole of one side of the cortical bone to near the cortical bone of the opposite side (Figure 14a, Supplementary Figure S16a,b), and the implantation materials were largely unchanged from their original form. In the implantation holes, thin regenerated bone extended from both sides of the hole to cover the implantation holes, but in the central part, the implantation materials were exposed outside the bone. The shape and staining characteristics of the implantation materials were similar to uHA(10)/PLGA at the left femoral metaphysis. Inside the bone, it was intermittently enveloped by thin fibrous and bone tissues, and in some parts of the sides, infiltration of cells, including macrophages and neutrophils, into the implantation materials was observed (Figure 14b, Supplementary Figure S16c,d). No clear positive findings were observed with OC immunostaining. Based on these observations, the condition of PLGA/uHA(10) was suggested to be similar to that of uHA(40) at 1 week post-implantation surgery. In the right distal femoral diaphysis, no obvious masses of the implantation materials were observed within the bone defects that were presumed to be implantation holes or within the bone marrow (Figure 14c, Supplementary Figure S16e,f). However, foreign materials accompanied with foam cells, which were similar to foreign materials accompanied with adipocytes observed in the bone marrow at the right distal femoral metaphysis, were observed (Figure 14d, Supplementary Figure S16g,h). Thus, it was suggested that phagocytosis of the implantation materials was occurring.

#### 2.4.2. Evaluation of the Left Femoral Bone Defect Area

**Figure 15:**
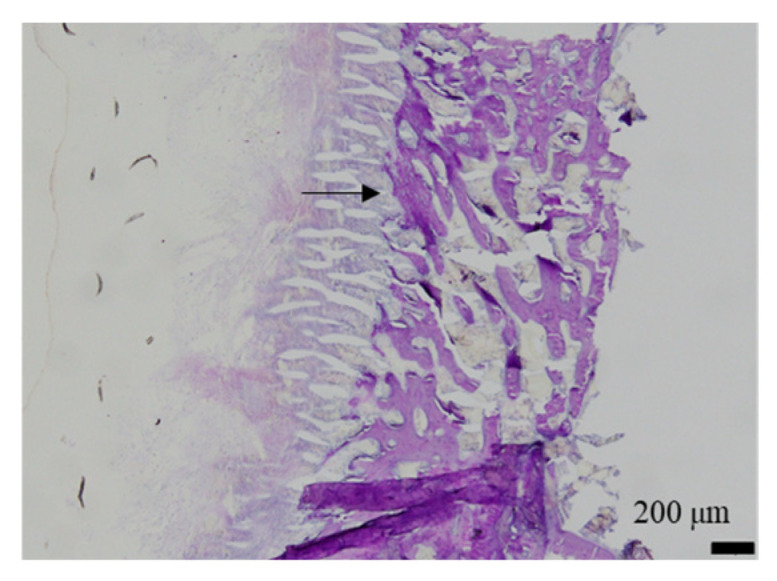
Representative histological image of bone defect area of the diaphysis in Rabbit-No. 1 at 1 week after implantation surgery stained by PAS (×40).

The histological evaluation results of the diaphysis (bone defect area) at 1 week post-implantation surgery are shown in Figure 15 (Supplementary Figure S17) (Rabbit No. 1). Regenerated bone, including some cartilage-like areas (Figure 15, Supplementary Figure S17a,b: arrows) accompanied by osteoid, covered the defect area and was thinner than the thickness of the cortical bone. Bone marrow tissue was not observed between the regenerated bones, and only a few mononuclear cells were observed. Thus, it was suggested that normal bone regeneration was taking place 1 week post-implantation surgery, and it provided an indication of the degree of bone regeneration at this stage.

**Figure 16:**
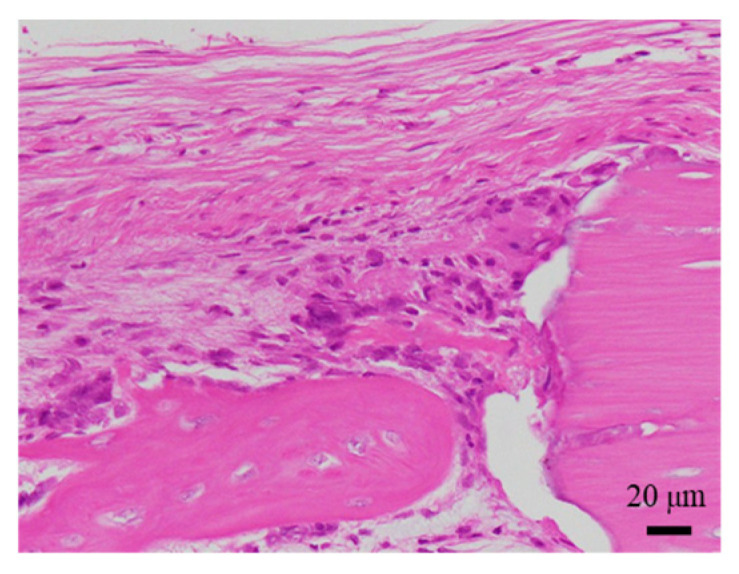
Representative histological image of bone defect area of the diaphysis in Rabbit-No. 4 at 4 weeks after implantation surgery (×100).

The histological evaluation results of the diaphysis (bone defect area) at 4 weeks post-implantation surgery are shown in Figure 16 (Supplementary Figure S18) (Rabbit No. 4). The implantation holes were filled with new bone, and the outer fibrous membrane tissue (periosteum) showed slight thickening (Figure 16, Supplementary Figure S18a–c). The newly formed bone extended almost horizontally and connected the cortical bone defect edge in the upper part of the defect area. Clear granulation tissue was not formed, but a few macrophages were observed in minor gaps between the cortical bone defect edge and the new bone (Figure 16, Supplementary Figure S18c). From these findings, it was suggested that normal processes of bone regeneration were progressing.

**Figure 17:**
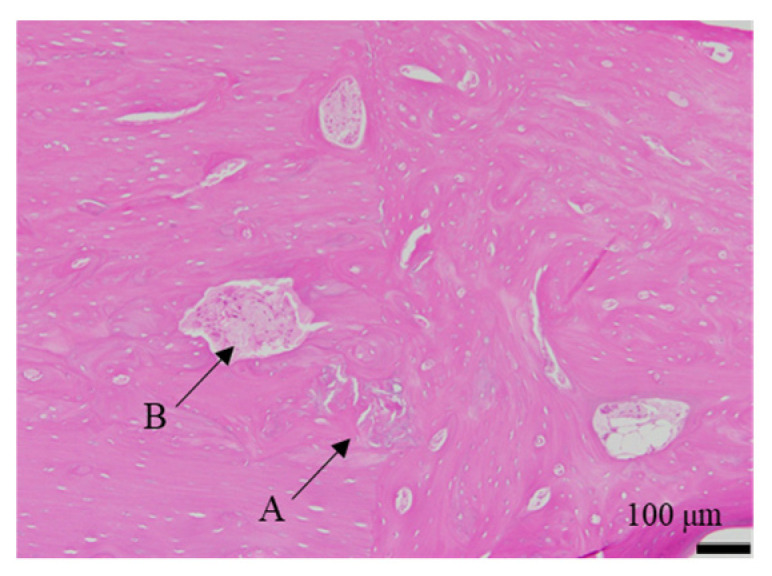
Representative histological image (HE) of bone defect area of the diaphysis in Rabbit-No. 7 at 6 weeks after implantation surgery (×100). A, foreign materials observed inside the newly formed bone; B, foreign materials observed outside the newly formed bone.

The histological evaluation results of the diaphysis (bone defect area) at 6 weeks post-implantation surgery are shown in Figure 17 (Supplementary Figure S19) (Rabbit No. 7). The overall view is shown in Supplementary Figure S19a. Slight structures resembling foreign materials were observed within the bone matrix of the cortical bone, and clusters of macrophages were observed within the lacunae (Figure 17, Supplementary Figure S19b: A,B). These structures resembling foreign materials are likely to be blood clots or tiny bone fragments resulting from damage to the surrounding tissues in the bone defect area. However, in this study, there is no particular noteworthy point to mention. PAS staining showed negative findings, but OC immunostaining (Supplementary Figure S19c) showed positive findings in the structures resembling foreign materials and the surrounding bone.

**Figure 18:**
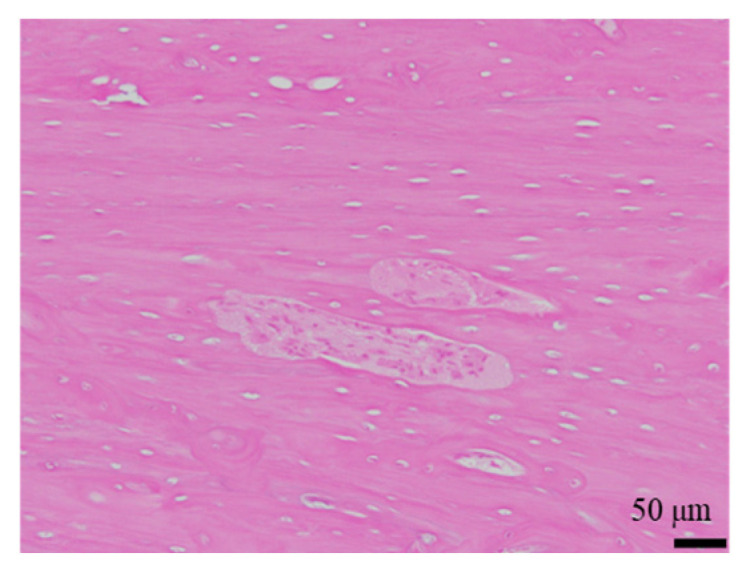
Representative histological image (HE) of bone defect area of the diaphysis in Rabbit-No. 8 at 9 weeks after implantation surgery (×200).

The histological evaluation results of the diaphysis (bone defect area) at 9 weeks post-implantation surgery are shown in Figure 18 (Supplementary Figure S20) (Rabbit No. 8). Although no evidence of increased new bone growth containing foreign materials, similar to what was observed in the distal part, was found within the bone marrow (Supplementary Figure S20a), clusters of macrophages were observed within the cortical bone (Figure 18, Supplementary Figure S20b). PAS staining revealed negative findings, but materials that were stained pale yellow with EVG staining (Supplementary Figure S20c) and showed positivity to OC immunostaining (Supplementary Figure S20d) resembling a foreign body were faintly observed. The material resembling a foreign body is believed to be similar to that observed in the specimen from 6 weeks after the implantation surgery. The overall condition appeared to be similar to that at 6 weeks post-implantation surgery, but it was thought that biological reactions were more advanced, such as bone regeneration.

**Figure 19:**
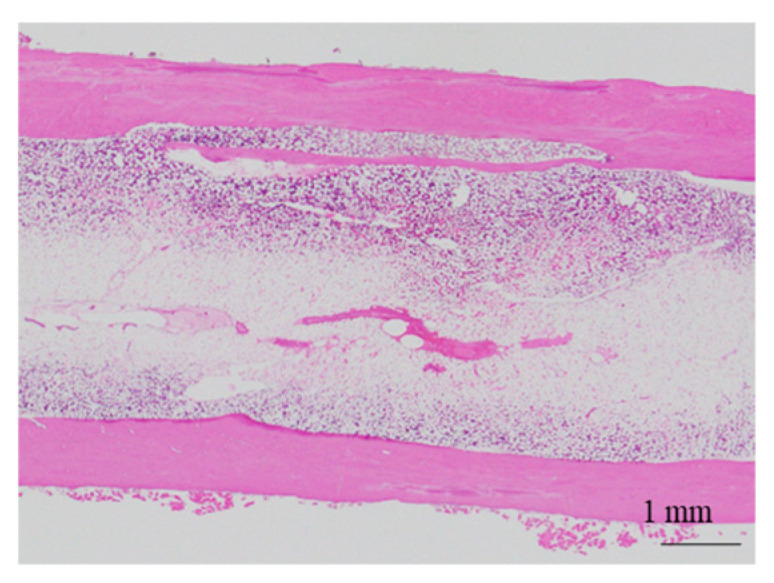
Representative histological image (HE) of bone defect area of the diaphysis in Rabbit-No. 3 at 48 weeks after implantation surgery.

The histological evaluation results of the diaphysis (bone defect area) at 48 weeks post-implantation surgery are shown in Figure 19 (Supplementary Figure S21) (Rabbit No. 3). Blood vessel distribution was observed in the central part of the bone marrow, with lower cell density around it. Near the cortical bone, there was a slightly higher cell density (Figure 19, Supplementary Figure S21). However, no noteworthy phenomena were observed, suggesting that bone regeneration was proceeding smoothly and was in the late stages of completion.

**Figure 20:**
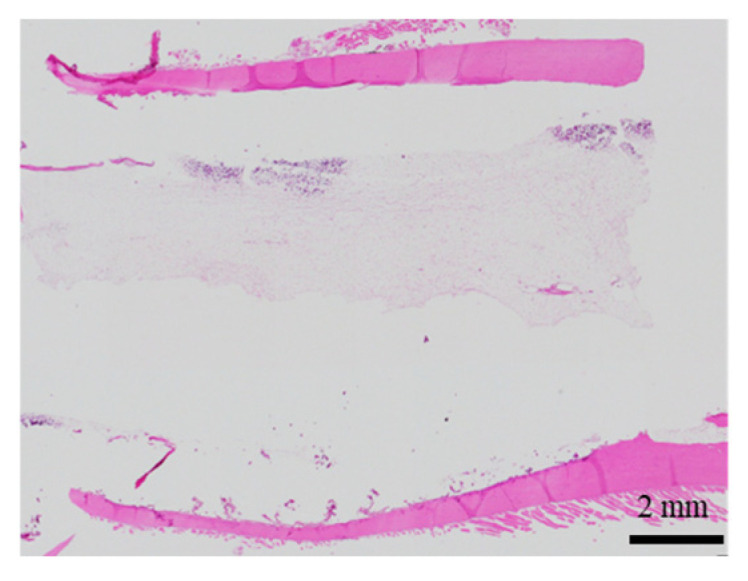
Representative histological image (HE) of bone defect area of the diaphysis in Rabbit-No. 6 at 48 weeks after implantation surgery.

The histological evaluation results of the diaphysis (bone defect area) of Rabbit No. 6 at 48 weeks post-implantation surgery are shown in Figure 20 and Supplementary Figure S22. There were no significant abnormalities observed in the bone tissue, and it appeared to be in a similar state to that of Rabbit No. 3, suggesting that the phenomena were reproduced.

## 3. Discussion

### 3.1. Animal Breeding Conditions

Regarding the management of the animals, there were no significant abnormalities observed in body weight changes, and considering the temporal changes in CRP (Figure 2), it can be assumed that, except for Rabbit No. 2, healthy and successful husbandry practices were maintained. CRP analysis values were elevated at 1 week post-implantation surgery (Rabbit No. 4, 6, 7, 8), which is likely attributed to the inflammatory response following surgery. However, it subsequently decreased after an initial rise (at 2 weeks post-implantation surgery), but at 4 weeks post-implantation surgery, it increased again in almost all specimens, albeit with some quantitative individual differences. This result, when considered in conjunction with the results of PET/CT and histological evaluations, is likely due to the high expression of macrophages aiming at removing the implantation materials, as it aligns with the timing of macrophage accumulation around the implantation materials. Subsequently, the inflammatory marker levels decreased and stabilized at low values, and in terms of the trend for the temporal changes, it remains similar to the accumulation status of macrophages around the implantation materials. On the other hand, it is considered that the accumulation of macrophages around the implantation materials themselves is reflected in the signal changes of PET/CT (FDG). Therefore, the early decrease in CRP levels compared with the temporal changes in FDG is due to CRP reflecting inflammation throughout the entire body and settling earlier at the systemic level, which is distinct from the changes in FDG that reflect localized alterations.

### 3.2. Evaluation of the Conditions Within the Femur Bone

Although the implantation site and the bone defect site were located in the femur diaphysis, here, we refer to them as “diaphysis” and “bone defect”, respectively.

At first, we tried to assess the presence of osteoclasts by TRAP/ALP double staining, but in this study, no clear positive reaction was observed with TRAP/ALP double staining because all samples had to be evaluated histologically after demineralization, which caused inactivation of the enzymatic reaction.

#### 3.2.1. Visual Evaluation of Temporal Changes in Implantation Materials

Visual evaluation included obtaining volume rendering images from CT scans. In the CT images of the diaphysis and the metaphysis, uHA(40) showed thin remnants or traces of the implantation materials at approximately 48 weeks post-implantation surgery. In contrast, β-TCP(40) had become mostly unrecognizable by around 24–36 weeks post-implantation surgery (Figure 3). This trend is consistent with the quantitative evaluation results of CT values as described later, and when comparing uHA(40) to β-TCP(40), it is considered that β-TCP(40) disappears from the bone faster than uHA(40). On the other hand, uHA(10), which initially contained a lower amount of calcium phosphate, showed almost no remnants by 36–48 weeks post-surgery. However, uHA(10)/PLGA still had noticeable remnants at 48 weeks post-surgery. This is likely related to the rate of polymer degradation. Although the degradation or disappearance of the polymer and calcium phosphate dissolution are believed to occur almost simultaneously and in parallel with the loss of calcium phosphate, the state of polymer degradation appeared to affect the ease of calcium phosphate disappearance, and this result provided the evidence that the state of polymer degradation affects the ease of calcium phosphate disappearance.

#### 3.2.2. Evaluation of Bone Defects

The internal structure of bone undergoes complex temporal changes in the surgical area. Therefore, in this study, we primarily focus on the simplest system, which is the bone defect in the diaphysis. The evaluation of bone defects using PET/CT was divided into two regions: the bone marrow region (Figure 6) and the cortical bone region (Figure 7).

**Figure 21:**
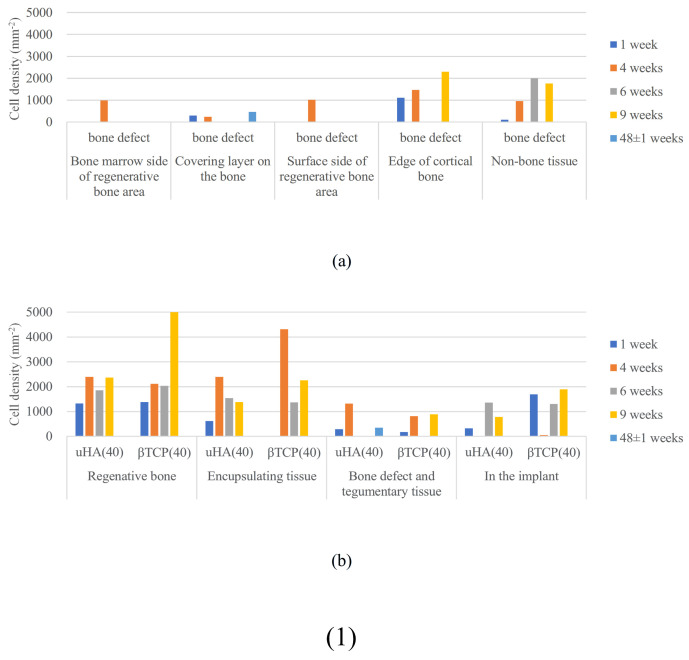

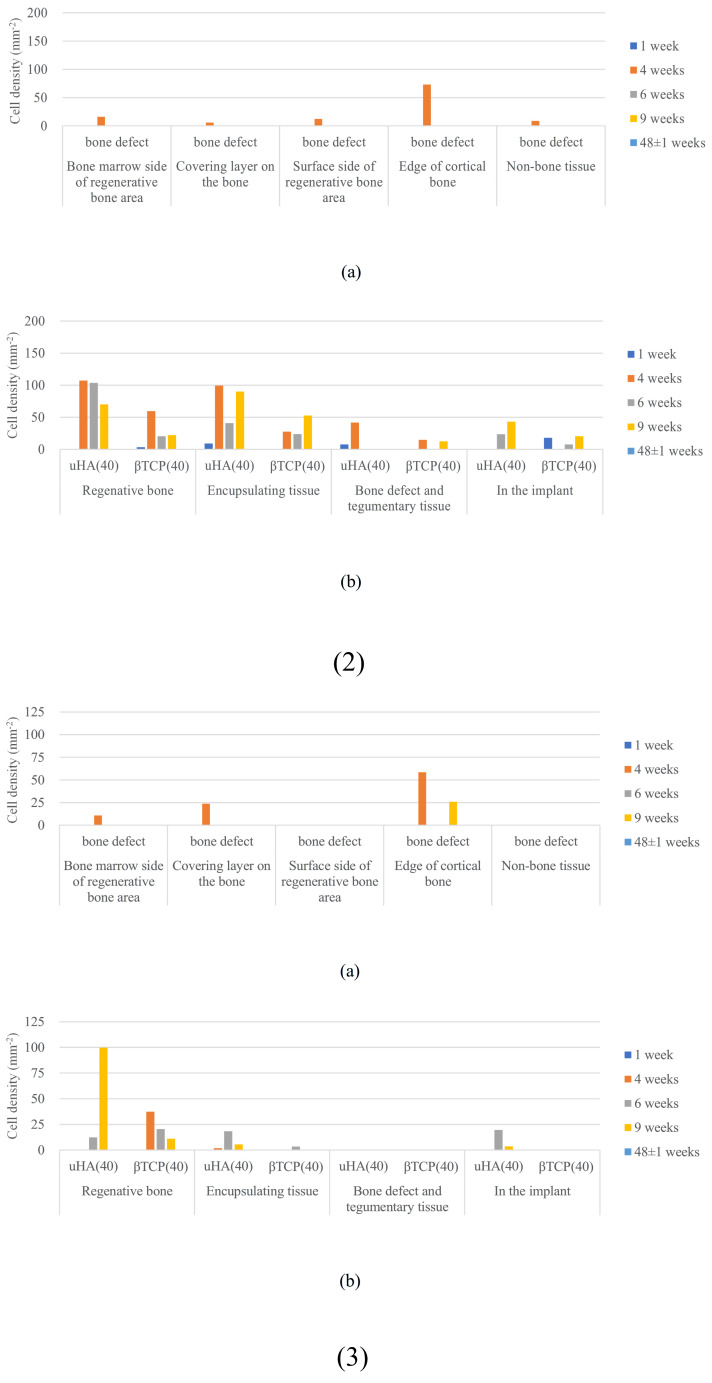

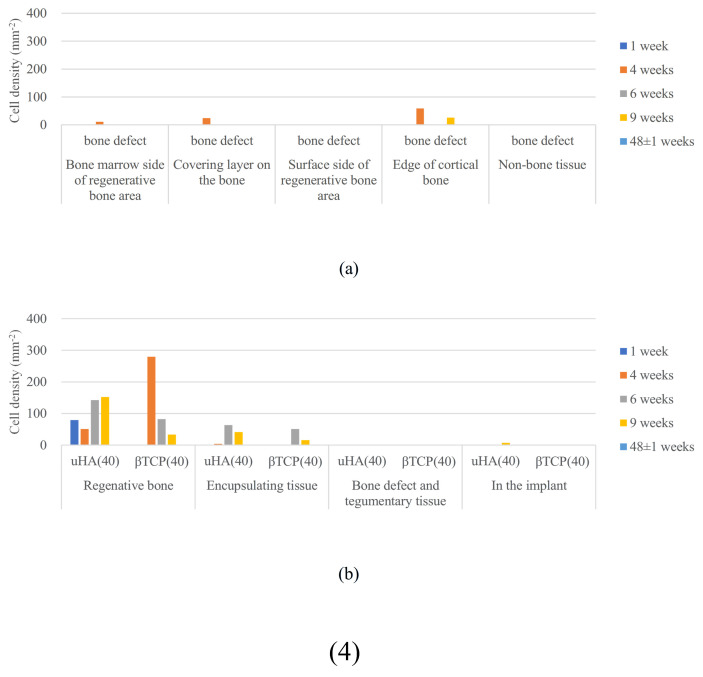
Quantitative histological evaluation around (**a**) the bone defect area in the diaphysis and (**b**) the implantation area in the diaphysis. (**1**), (**2**), (**3**), (**4**) show the number of infiltrating macrophages, giant cells, osteoclasts and osteoblasts, respectively.

First, concerning the bone marrow region, CT values in all samples started around 0 HU at the time of surgery (0 W) and peaked at 1–2 weeks. However, the maximum values were relatively low, typically in the tens of HU. This result reflects the fact that, immediately after creating the bone defect, there was no bone tissue regeneration, and no implantation materials were present, resulting in CT values around 0 HU. Over the course of several weeks, the healing process progressed. The bone marrow region, being composed of soft tissue, did not show the high CT values typical of cortical bone, and it maintained stable values around those mentioned above [46]. Furthermore, the subsequent decrease in CT values after reaching a peak is likely due to the fact that, during the bone regeneration transitional period, cellular activity was intense, leading to the formation of excessive bone-related tissues [47,48,49]. This temporarily resulted in slightly higher values than those seen during the final stable period, and it is thought that this excess bone-related tissue was subsequently eliminated, leading to the decrease in CT values. Ultimately, the stable values observed are similar to those seen during the stable phase of bone regeneration following the implantation of β-TCP(40) and uHA(10) in the diaphysis (Figure 4, Figure 5, Figure 6 and Figure 7, uHA(40), implanted in the diaphysis, is thought to not have completely disappeared by the time of 48 weeks post-implantation surgery and thus exhibits higher CT values than CT values of normal bone marrow [50,51]). These stable values were also similar to those of the untreated bone marrow region in this study. NaF, which is an indicator of new bone, showed a maximum value at 1 or 2 weeks post-implantation surgery and then decreased monotonically, slightly preceding and synchronizing with the change in CT values. This suggests that bone regeneration begins shortly after the implantation surgery. Furthermore, it can be understood that there was an excessive phase of bone regeneration during which an excess of bone-related tissues was also generated. On the other hand, the temporal changes in FDG also synchronized with the changes in CT value, but the timing of the peak coincided with the CT values or slightly lagged behind. In the histological evaluations performed at 1, 4, 6, 9, and 48 weeks post-implantation surgery, macrophages were predominantly observed at 4 weeks post-implantation surgery along with the accumulation of multinucleated giant cells and osteoclasts (Figure 21(1a),(2a),(3a)). This suggests that FDG reflects the accumulation of these macrophage-like cells. This seems to support the previously mentioned idea that phagocytic cells accumulated in order to eliminate excess tissue regenerated during periods of active cell activity [48].

The results in the cortical bone region are described. CT values in all specimens began to rise from around 0 HU at 0 weeks post-implantation surgery, reached their peak at 6 weeks post-implantation surgery, and then gradually declined until around 12 weeks post-implantation surgery, after which they stabilized. This result is consistent with the trends observed in the bone marrow region. However, the CT values during the stable period were in the range of 1000–1500, which is in line with CT values typically reported for cortical bone [52,53,54], suggesting that the healing of cortical bone was nearly complete. This trend was observed consistently in all specimens. The phenomena that the peak of NaF precedes the peak in CT values can be considered from the results of Rabbit No. 6 and 8, as shown in Figure 7. The peak is estimated to occur around 2–3 weeks post-implantation surgery. For Rabbit No. 3, the maximum values are observed at 2 weeks post-implantation surgery, with a subsequent steady decrease, although PET/CT measurements were not conducted at 1 week post-implantation surgery. Therefore, it can be inferred that, while there is no clear peak, the maximum value is observed at 2 weeks post-implantation. It is thought that this result reflects that there is a time lag between the onset of bone regeneration and its maturation into bone tissue [55,56,57,58,59,60,61,62,63,64,65,66]. This observation holds true for the bone marrow region as well. The delayed timing of the peak or maximum CT values in the cortical bone region compared with the bone marrow region can be attributed to the fact that the maturation of cortical bone requires a process of calcification, which takes more time compared with the maturation in the bone marrow region. On the other hand, FDG in the cortical bone region peaked at 3–4 weeks post-implantation surgery and then gradually decreased, stabilizing around 0 Bq/cc/Image Units from 12 weeks post-implantation surgery onwards (Figure 7). Unlike the bone marrow region, in this aspect, FDG showed an earlier peak compared with the peak in CT values. However, similar to the bone marrow region, this suggests that, during the period of active bone regeneration, phagocytic cells were beginning to remove excess bone-related tissues that may have been formed excessively. On the other hand, the fact that FDG peaks in both the cortical bone region and the bone marrow region at roughly the same time indicates that the accumulation timing of macrophages and other cells involved in the response during bone regeneration is not significantly different between these two regions. This observation indicates that bone regeneration and the removal of excess bone-related tissues, as suspected, are occurring concurrently. Taking into consideration the overall temporal changes in CT values, FDG, and NaF, it can be inferred that bone regeneration begins at an early stage after implantation surgery, at the latest, even earlier than 3 weeks post-implantation. During this period, both bone regeneration and the removal of excessive bone tissue occur simultaneously. Until approximately 6 weeks post-implantation, bone regeneration is predominant, subsequently while bone tissue removal becomes dominant until around 12 weeks post-surgery. After this point, it is presumed that a state of equilibrium is reached between bone regeneration and bone resorption. Furthermore, the behavior where CT values appear to overshoot around 6 weeks post-implantation suggests that, during the period of active bone regeneration, excess bone-related tissue is being generated and subsequently removed.

#### 3.2.3. Evaluation of the Diaphysis

Similar to the evaluation of the bone defect area, the diaphysis was also assessed separately for the bone marrow region (Figure 4) and the cortical bone region (Figure 5). As mentioned earlier in the “(2) Evaluation of Bone Defects” section, based on the results of histological evaluation, FDG is primarily reflective of the behavior of macrophage-related cells (macrophages, multinucleated giant cells, etc.) [67,68,69,70,71,72,73,74,75,76,77,78,79,80,81,82,83,84,85], and it is considered to mainly reflect the behavior of macrophages, considering their numbers and temporal changes. Furthermore, while NaF is widely used as an indicator of new bone formation [86,87,88,89,90,91,92,93], it was used similarly in this study as an indicator of new bone, and the experimental results were consistent with this usage.

First, regarding the bone marrow region, as observed in the histological findings, uHA(40) remained in the long term. After 12 weeks post-implantation surgery, the CT values had decreased compared with immediately after implantation, but even at 48 weeks post-implantation surgery, they still showed approximately half of the initial CT values. In contrast, for β-TCP(40), the CT values had reached around 0 by 12 weeks post-surgery, indicating that it had almost disappeared. Considering that there were no significant quantitative differences in the temporal changes in FDG between the two and based on the relatively large masses observed in the histological findings for uHA(40) (Figure 13a), it is suggested that the persistence of uHA(40) was not due to differences in the accumulation of phagocytic cells but rather because uHA(40) was in a less phagocytosed state compared with β-TCP(40). Furthermore, since the accumulation of multinucleated giant cells was generally more prominent in the uHA(40) implantation area compared with the β-TCP(40) implantation area (Figure 21(2b)), it can be speculated that, in the case of uHA(40), macrophages were unable to fully address removal of implantation materials, and multinucleated giant cells appeared to try to deal with it. Even though they gathered, they could not fully resolve the situation, resulting in the long-term presence of the implant material. These observations provide further evidence that the mechanism of disappearance of implantation materials is due to cellular phagocytosis, and the primary actors in phagocytosis are macrophages rather than osteoclasts. On the other hand, for the uHA-based implantation materials with lower uHA content, uHA(10) (Rabbit No. 6), it was almost completely absorbed around 12–24 weeks post-implantation surgery, similar to β-TCP (Figure 4(3)). The only difference between these two is the uHA content, whether it is 40% or 10%. Furthermore, after the start of breakdown of implantation materials (which began around 4 weeks post-implantation surgery, with significant breakdown occurring particularly after 6 weeks), there were no large foreign materials derived from the implantation materials, as observed with uHA(40). Specifically, at 48 weeks post-implantation surgery, no implantation materials of the size seen with uHA(40) were observed. In other words, it can be inferred that the uHA content affects the aggregation of uHA during the disappearance process, making it more prone to aggregation and difficult for macrophages and giant cells to process due to its size. Furthermore, in the case of the bone defect area, the CT value initially increased from 0 HU, peaked around 2–3 weeks post-implantation surgery, and then began to decrease, stabilizing at around 12–24 weeks post-implantation surgery (Figure 6). On the other hand, in the diaphysis where the implantation materials were placed, there was some variation, but it generally started above 1000 HU and exhibited a mostly monotonous decrease, stabilizing at around 12–24 weeks post-implantation surgery (Figure 4). The initial high CT values are indeed due to the presence of the implantation materials, but it is interesting that the time it takes to stabilize at a certain value appears to be roughly the same regardless of the presence or absence of the implantation materials (Figure 4 and Figure 6). In other words, in the bone marrow region, the time required for wound healing and removal of implantation materials appears to be similar. This implies that the removal of the implantation materials and wound healing are completed at approximately the same time. On the other hand, in terms of PET signal, both FDG and NaF showed a shift in the timing of their peaks, occurring later than for the bone defect region (Figure 4 and Figure 6). This phenomenon, where the peaks shift to a later time period due to the presence of the implantation materials, can be understood as a result of the initial limited access of phagocytic cells, such as macrophages and osteoblasts, involved in bone regeneration to the interior of the implantation materials. However, despite the shift to a late period in peak timing due to the presence of the implantation materials, the fact that bone regeneration and wound healing occur at the same time is likely because there is a significantly higher number of cells, including macrophage-lineage cells and cells involved in bone regeneration, gathered at the implantation site. This can be inferred from the fact that the signal values for FDG are more than twice as high and for NaF are more than four times as high in the implantation area of the diaphysis compared with the bone defect region (Figure 4 and Figure 6). Regarding FDG, the higher signal values support the idea that the biological response (removal) to the implantation materials by macrophage-lineage cells is more active. As for NaF, both uHA and β-TCP have been reported to have osteoconductivity effects [94,95,96,97,98,99,100,101,102,103,104], and these effects are significant, potentially guiding more cells involved in bone regeneration, primarily osteoblasts. However, for uHA(10), the peak value of NaF was lower than the maximum value in the bone defect. This could be due to the lower density of uHA, which might have led to a quantitatively reduced bone conductivity in the entire region. Nevertheless, around 4–6 weeks post-implantation surgery, when cells began invading the implantation materials, the NaF value in regions with implantation materials were more than twice as high as those in the bone defect region and sustained a more than twice as high level for more than 2 weeks. This indicates that the number of cells involved in bone tissue removal and bone regeneration, which enter the implantation materials, is higher when the implantation materials are present. Consequently, it can be assumed that uHA(10) followed a similar temporal result to β-TCP(40) due to these factors.

Next, the results for the cortical bone region are described. In the bone defect region, the CT values started at 0 HU, increased toward 6 weeks post-implantation surgery, and then decreased, stabilizing around 12 weeks post-implantation surgery (Figure 7). In the diaphysis region, except for the fact that the CT values started at approximately 1000 HU, the trend was generally similar to that observed in the bone defect region and in cases with only the polymer (PDLGA) (Figure 5, Figure 7 and Figure 8). The initially high CT values were due to the presence of the implantation materials. However, similar to the bone marrow region, the subsequent temporal changes in CT values in the cortical bone region were similar regardless of the presence of the implantation materials. This suggests that, in the cortical bone region, the disappearance of the implantation materials and wound healing are almost completed simultaneously. However, the final CT values are higher than in the bone marrow region due to the regeneration of cortical bone. The variability in these values may be attributed to the use of cylindrical ROIs that may not perfectly match the actual shapes, leading to potential errors. On the other hand, in the diaphysis, both FDG and NaF PET signals in the cortical bone region in the case of implantation exhibited a trend of delayed peaks compared with the bone defect case, which is similar to the result in the bone marrow region (Figure 4, Figure 6 and Figure 8). The likely cause for this phenomenon is considered to be similar to the mechanism in the case of the bone marrow region.

#### 3.2.4. Study Limitations

In this study, it was challenging to allocate a sufficient number of animals to each time point in advance because systematic methodologies that integrate functional imaging with histopathological evaluation for assessing the in vivo resorption of implanted materials have not yet been established. Moving forward, study designs incorporating increased numbers of animals—thereby enabling more systematic and statistically adequate analyses—will be an important direction for future investigation.

## 4. Materials and Methods

### 4.1. Animals and Implantation Samples

#### 4.1.1. Experimental Animals

We used male rabbits (Japanese White (JW), purchased from Oriental Yeast Co., Ltd., Tsukuba, Japan) as experimental animals. Individuals with similar body weight and age at delivery (body weight 2.6–2.9 kg, age 13 weeks: Supplementary Table S1) were obtained in three separate batches to accommodate the experimental schedule.

#### 4.1.2. Breeding and Health Monitoring

To ensure the stability of the rabbits’ health, they were transported to Hamamatsu University School of Medicine a few days before the implantation surgery and were housed there. Food and water were provided to the rabbits for free access, but they were fasted for over 12 h starting from the day before the PET/CT measurements. The body weight of the rabbits was measured before each PET/CT measurement day.

#### 4.1.3. Implantation Samples

The implantation samples were non-porous composites designed for observing in vivo dynamics and were composed of a copolymer of dl-lactide and glycolide (PDLGA) and calcium phosphates (Table 1). For calcium phosphate, we selected two types: uHA (unsintered hydroxyapatite) [39,40,41,42,43] and β-TCP (β-tricalcium phosphate) [44,45], well-known in this research field. The weight ratio of calcium phosphate was set at 40% for both uHA and β-TCP samples (uHA(40) and β-TCP(40), respectively). Furthermore, to assess the influence of the amount of calcium phosphate, we prepared samples with a weight ratio of 10% calcium phosphate using uHA (uHA(10)). To evaluate the impact of the hydrolysis rate of the polymer, we also prepared samples with a weight ratio of 10% calcium phosphate using uHA, but in this case, we used a slow-hydrolyzing PLGA, copolymer of l-lactide and glycolide (uHA(10)/PLGA). The two kinds of calcium phosphate used had a secondary particle size of 3–5 µm and were purchased from Taihei Chemical Industrial Co., Ltd. (Osaka, Japan). Regarding PDLGA, for considerations of degradability in the design of the study period, we used a copolymer of dl-lactide (LA) and glycolide (GA) in a 50:50 ratio, with a weight-average molecular weight (Mw) of 10.4 × 10^4^. Sample preparations were conducted using the same methods described in the literature [105]. The materials were then machined into the specified shape and dimensions, followed by sterilization via electron-beam irradiation at a dose of 20 kGy.

#### 4.1.4. Allocation of Samples

Here, we provide information about the allocation of implanted samples to experimental animals. Bone implantation was performed at two sites, specifically, the femoral metaphysis and the femoral diaphysis. The femoral diaphysis is a region rich in cancellous bone, whereas the femoral diaphysis region has a lower density of cancellous bone. Furthermore, on one side of the diaphysis (left side), we also created a bone defect site (refer to Section 4.1.5). This defect size was the same as the opening size when implanting implantation samples. The details regarding the allocation of samples to implant sites, observation period, and the timing of implantation surgeries (which were conducted in three separate sessions) are provided in Table 2.

#### 4.1.5. Implantation Surgery

The implantation surgeries were conducted in three separate sessions, as indicated in Table 2. The surgical procedures for each session were identical.

##### Creation of Bone Defect (Implantation) Model

A mixture of Ketamine Hydrochloride (Ketalar, intramuscular injection, 500 mg, 50 mg/mL) and Xylazine (Seralcatal, 2% injection, 20 mg/mL) in a 1:1 (*v*/*v*) ratio was administered intramuscularly (0.5 mL/kg) to induce anesthesia (Table 3). Maintenance anesthesia was then performed using inhaled Isoflurane. The surgical fields were shaved around both knee joints, and the areas were disinfected with veterinary iodine solution and ethanol for disinfection. The skin was incised, and the periosteum was separated or incised from the muscle to expose the bone while minimizing damage to the muscles. In the central region of the diaphysis, a non-penetrating hole was created gradually with an electric drill, expanding to approximately ϕ4 × 7 (mm) in size, reaching the inner cortical bone surface on the opposite side. Similarly, bone defects of approximately ϕ3 × 5 (mm) were created at two locations on the left and one location on the right in the diaphysis. Both defect sizes were smaller than the critical defect size. During the procedure, physiological saline (Fuso Pharmaceutical Industries, Ltd., Osaka, Japan), containing Enrofloxacin (Baytril 2.5% injection for dogs and cats, Bayer Yakuhin, Ltd., Osaka, Japan), was intermittently applied to the surgical site while flushing away bone fragments. This was done to prevent heat-induced damage to bone tissue. The periosteum of the defect area was removed, and after thorough cleaning of the surgical site, the samples were implanted into the bone defect. The muscle layer, subcutaneous tissue, and skin were sutured. Implantation was performed on both the left and right sides, with the implantation day considered as Day 0. The details of the samples and implant sites are provided in Table 2.

##### Postoperative Management

Postoperatively, pain relievers and antibiotics were administered once a day for more than three days, including the day of the procedure. The pain reliever used was Buprenorphine at a ratio of 0.02 mg/0.1 mL/kg, and antibiotics were administered as Enrofloxacin at a ratio of 10 mg/0.4 mL/kg, both administered intramuscularly.

#### 4.1.6. Inflammation Assessment

To monitor the inflammatory state of the rabbits, blood samples were collected at a frequency of once per month, and C-reactive protein (CRP) measurements were performed using the enzyme-linked immunosorbent assay (ELISA) method. The CRP ELISA kit used was the abcam (ab157726) C-Reactive Protein (PTX1) Rabbit ELISA Kit from Abcam. A microplate reader, specifically the Synergy H1 (Read Speed: Normal, Delay: 100 ms, Measurements/Data, Point: 8, Actual Temperature: 22.3), was used. The serum from the rabbits to be measured was divided into two tubes, and CRP measurements were conducted. The average value of these measurements was used for evaluation.

Blood was collected from the rabbits under anesthesia, and the venous route was accessed through the ear. Approximately 0.5 mL of blood was collected for CRP testing. The collected blood was allowed to stand at room temperature for approximately 30 min. Afterward, it was subjected to centrifugation (1400 G, 20 °C, 10 min), and the serum was separated. The serum was then frozen and stored for subsequent batch measurements.

### 4.2. PET/CT Measurement

The temporal changes related to implantation materials and bone regeneration were evaluated using CT scans. Furthermore, the temporal changes in cellular gathering and dispersion primarily focused on macrophages were assessed using FDG, which had been also used in diseases such as cancer, etc. [67,68,69,70,71,72,73,74,75,76,77,78,79,80,81,82,83,84,85,107,108,109,110,111,112,113,114,115,116,117], and the temporal changes in the cell histological aspects related to bone regeneration were evaluated using NaF [79,80,81,82,83,84,85,86,87,88,89,90,91,92,93].

#### 4.2.1. CT Measurement

On the evening before the PET/CT measurement, the rabbits were fasted for more than 12 h. Body weight was measured before the CT measurement. The rabbits were anesthetized according to the conditions specified in Table 3. After inducing anesthesia, they were positioned in a prone position on a dedicated rabbit bed and immobilized with tape or similar means to prevent movement. They were then dressed in diapers and an anesthesia mask before the CT measurement. The measurements were performed using the GE Bright Speed 16 ch (Supplementary Figure S23a). The measurement conditions were set to helical mode, tube voltage/tube current of 100 kV/Auto, and a slice pitch of 0.625 mm.

#### 4.2.2. PET Measurement

After the CT imaging, the rabbits were transported while remaining on the fixed bed to the PET apparatus, where the positioning for PET imaging was carried out. While observing the rabbit’s respiratory status, the anesthesia was switched to isoflurane inhalation anesthesia (Isoflurane: 1.25%, oxygen: 0.5 L/min, air: 0.5 L/min). The isoflurane concentration started at 0.5% and was increased to 1.25% as needed.

Under anesthesia, ^18^F-FDG (hereafter referred to as FDG) was administered through the rabbit’s ear vein. Forty-nine minutes after administration, a PET scan of the lower limbs was initiated and measured for 10 min. After waiting for two days until the FDG signal was no longer detectable in the rabbit’s body, PET/CT measurements were performed using ^18^F-NaF (hereafter referred to as NaF), following the same procedure as with FDG. The measurements were conducted between 109 min and 119 min following NaF administration. The measurement conditions and timing for both FDG and NaF administration were determined through preliminary experiments conducted before the start of this study The PET device used was a custom-made device from Hamamatsu University School of Medicine (Supplementary Figure S23b). The preparation and administration of the probe were conducted as follows: since the half-life of ^18^F is short, approximately 108 min [118,119,120,121,122,123,124,125,126], the ^18^F used for FDG and NaF was produced on-site using the cyclotron at Hamamatsu University School of Medicine for each experiment. FDG and NaF were synthesized using an automated synthesizer. FDG and NaF were diluted with saline and administered to the rabbits at a dose of 111 MBq (3 mCi) per 0.5 mL (PET/CT measurement workflow: Supplementary Figure S24).

### 4.3. Data Analysis

**Figure 22:**
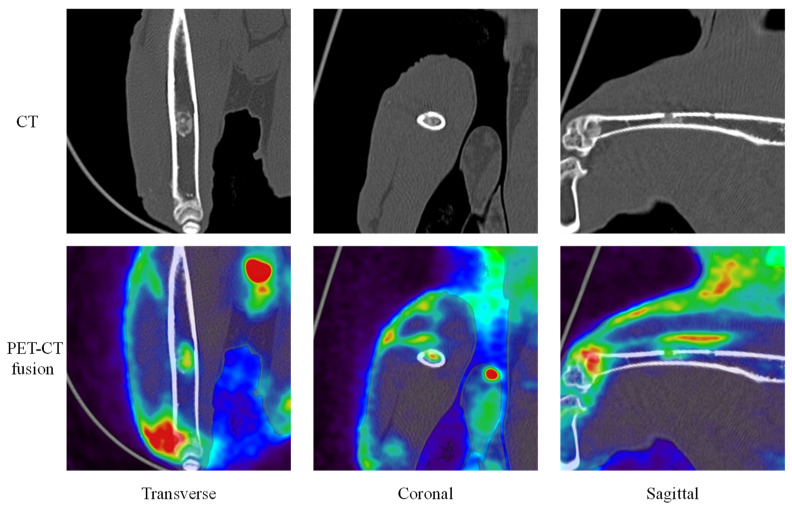
Example of fusion of CT data and PET images. With Transverse, Coronal and Sagittal views, the PET images are superimposed on the CT image in all directions. As probe accumulation increases, the display color in the PET images shifts from green to yellow and subsequently to red.

Data analysis involved the import of DICOM data from CT images acquired with the GE CT device and the raw data from PET images acquired with the PET device into the analysis software AMIDE (an open-source software, version 1.0.5). In AMIDE, CT images were fused with PET signals. The procedure involved fusion (registration) of CT images of the same individual in chronological order for each part and then fusion of PET images with the individual CT data (an example is shown in Figure 22). Subsequently, while confirming the CT images, regions of interest (ROIs) were defined for each implant site as well as for bone defect areas. Within these ROIs, CT values and standardized uptake values (SUVs) corrected for radioactive decay were calculated.

The size of the ROI for the bone marrow region was set at ϕ3 × 4 (mm) at the metaphysis and ϕ2.5 × 2.5 (mm) at the diaphysis. In this case, slightly smaller ROIs than the implants were set to accurately assess the temporal changes in CT values, ensuring that the cortical bone region was not included. On the other hand, for the cortical bone region, the ROI size was set at ϕ4 × 1 (mm) at the metaphysis and ϕ3 × 1.2 (mm) at the diaphysis. In this case, the ROI diameter was adjusted to match the size of the implant, and the thickness was set to the maximum value just below the thickness of the cortical bone after checking all CT data. Regarding the metaphysis, there was some variability in thickness settings in CT images due to the posture of the rabbits and the degree of knee flexion. Therefore, a slightly smaller thickness ROI was set for the metaphysis compared to the diaphysis. The ROI diameter was adjusted to match the size of the implant to evaluate the temporal changes in cortical bone regeneration. Using these results, we evaluated the temporal changes in each implant site and performed quantitative comparisons between individuals. Furthermore, when handling CT images for observing the condition and position of the implants, we also utilized CT analysis software (ZIO Cube Ver. 1.0.2.0, provided by Zaiosoft Inc., Tokyo, Japan) as needed. In particular, this software was used for constructing volume rendering images as described later.

### 4.4. Histological Evaluation

The implants and the surrounding tissues were excised from the experimental animals. After excision, they were fixed in 10% NBF and then replaced with 70% ethanol. For each experimental animal, a total of five samples were collected, including the diaphysis and metaphysis of both femurs (distal) and the diaphysis of the left femur (proximal).

#### 4.4.1. Sample Preparation and Staining

Each specimen was trimmed using a DIAMOND SAW V-19 (LUXO Corporation, Nagoya, Aichi, Japan). In the cases where fixation of bone marrow and other components was insufficient, re-fixation was performed for 2 days in 10% neutral buffered formalin (NBF) and then subjected to 6 days of defatting treatment in 70% ethanol. After 44 days of decalcification in a decalcification solution containing 10% ethylenediaminetetraacetic acid (EDTA) and 1% zinc sulfate in a ratio of 100 mL:0.4 mL, the specimens were cut to create specimen preparation surfaces. Dehydration and paraffin infiltration were then performed using Tissue-Tek VIP 6 AI (Sakura Finetek Japan Co., Ltd., Tokyo, Japan) to prepare paraffin-embedded blocks.

Slices were prepared from the paraffin blocks using a sliding microtome LS113 (Yamato Kohki Industrial Co., Ltd., Saitama, Japan). Seven slices were prepared and subjected to various staining procedures, including hematoxylin–eosin (HE) staining, Elastica-Van Gieson (EVG) staining [115,116,117,118,119,120,121], Periodic acid-Schiff (PAS) reaction [122,123,124,125,126,127,128,129], Tartrate-resistant acid phosphatase/Alkaline phosphatase (TRAP/ALP) double staining, and immunostaining for osteocalcin (OC) [130,131,132,133,134,135,136,137]. For TRAP/ALP double staining, the TRAP staining kit (#294–67001; FUJIFILM-Wako Pure Chemical Corporation, Osaka, Japan) was used. For OC immunostaining, anti-osteocalcin mouse monoclonal antibody, Clone: OC4–30 (GTX13418; GeneTex, Irvine, CA, USA), was used at a concentration of 0.1 µg/mL. The specimens were treated with Proteinase K (S3004; Agilent Technologies, Santa Clara, CA, USA) at room temperature for 2 min, followed by an overnight reaction at 4 °C. The secondary antibody used was horse radish peroxidase (HRP)-conjugated anti-mouse IgG goat polyclonal antibody (HISTOFINE #424134; Nichirei Bioscience Inc., Tokyo, Japan). Visualization was performed using 3,3′-diaminobenzidine, tetra-hydrochloride (DAB), followed by counterstaining with hematoxylin.

#### 4.4.2. Observation and Image Capture

The stained specimens were observed using an optical microscope BX-43 (OLYMPUS Corporation, Tokyo, Japan). Macroscopic images of tissues were captured using an SX720 HS camera (Canon Inc., Tokyo, Japan), and microscopic images were taken using the DP-22 imaging device (OLYMPUS). Whole-area images of the implantation site (magnifying images) were created using the Multiple Images Alignment (MIA) function of CellSens (OLYMPUS) from 40× images captured with the DP-22.

## 5. Conclusions

In order to clarify the absorption mechanism of biodegradable materials used as artificial bones and bone grafts, we prepared composites made from calcium phosphate and polymers for implantation in experimental animals and evaluated their in vivo changes and accompanying cell behavior over time using PET/CT. For making these composites, two typical types of calcium phosphate, β-TCP and uHA, were used as the calcium phosphate, and a copolymer of PDLGA and PLGA was used as the polymer. The differences in the phenomena caused by the different calcium phosphate content were also evaluated over time. Furthermore, histological evaluation of the characteristic periods in these results revealed the types of cells involved in the phenomena. The results are summarized below:

(1) The absorption mechanism of the biodegradable materials used in this study in the living organism was suggested to be mainly phagocytosis by macrophages.
(2) The disappearance rate within the living organism is faster for β-TCP(40) compared with uHA(40).
(3) The above-described 2 supports the consideration that uHA has a tendency to aggregate more readily in vivo than β-TCP, and this mechanism implies that it is less susceptible to phagocytosis by macrophages or multinucleated giant cells.
(4) Related to the above-mentioned 2 and 3, uHA(10), having a lower proportion of uHA, is not prone to aggregation and exhibited a similar disappearance result to β-TCP(40).
(5) From clinical perspectives, there are many cases where bone substitutes are required. However, the presence of implantation materials can delay bone regeneration, making it important to optimize the type and amount of polymer and calcium phosphate used.

To avoid misunderstanding, we would like to emphasize the following points here. Despite the delayed healing, the use of the materials in this study as bone replacement materials is important in clinical practice for several reasons. Namely, these materials prevent post-healing problems such as pseudoarticular joints by preventing soft tissue invasion into the trunk during the healing period, prevent secondary damage such as severe fractures in bone defects, provide mechanical support to the bone defect and promote maturation of regenerated bone with slight loading on the affected area relatively early, resulting in better quality, promoting bone regeneration, preventing complications such as infection, etc.

## Figures and Tables

**Table 1:** Implantation material and size and site. Abbreviation: uHA is unsintered hydroxyapatite, β-TCP is β-tricalcium phosphate, PDLGA is poly-dl-lactide-*co*-glycolide, PLGA is poly-l-lactide-*co*-glycolide.

	Density (g/cm^3^)	Calcium Phosphate Weight Ratio (%)	Site	Size (mm)
uHA(40)	1.70	36.2	metaphysis diaphysis	*ϕ*4 × 7 *ϕ*3 × 5
β-TCP(40)	1.71	38.5	metaphysis diaphysis	*ϕ*4 × 7 *ϕ*3 × 5
uHA(10)	1.46	9.0	diaphysis	*ϕ*3 × 5
uHA(10)/PLGA			diaphysis (Rabbit No.6)	*ϕ*3 × 5

The shape, density, and weight ratio of calcium phosphate in the implanted samples are presented in Table 2. For sample preparation, as previously reported, we produced a dense body by compressing a composite fibrous material composed of calcium phosphate and polymer, which was prepared using the dry spinning method [105,106]. This dense body was machined to the final shape. The machined samples underwent electron beam sterilization before implantation. The Mw after sterilization was measured and found to be 7.1 × 10^4^.

**Table 2:** Implant characteristics. uHA(10) and uHA(40) are composites with PDLGA, where the calcium phosphate type is uHA and the calcium phosphate weight ratio is 10% and 40%, respectively. β-TCP(40) is a composite with PDLGA, where the calcium phosphate type is β-TCP and the calcium phosphate weight ratio is 40%. uHA(10)/PLGA is a composite with PLGA, where the calcium phosphate type is uHA, and the calcium phosphate weight ratio is 10%.

Rabbit No.	Metaphysis		Diaphysis				Breeding Period	Implantation
	Left	Right	Left (Distal)	Left (Proximal)	Right (Distal)	Right (Proximal)		
1	uHA(40) *ϕ*4 × 7 mm	β-TCP(40) *ϕ*4 × 7 mm	uHA(40) *ϕ*3 × 5 mm	defect *ϕ*3 × 5 mm	β-TCP(40) *ϕ*3 × 5 mm	–	1 w	1st
2							48 w	1st
3							48 w	1st
4							4 w	2nd
5	No treatment (reserve animal, not used)							
6	uHA(10)/PLGA *ϕ*4 × 7 mm	uHA(10) *ϕ*4 × 7 mm	uHA(10)/PLGA *ϕ*3 × 5 mm	defect *ϕ*3 × 5 mm	uHA(10) *ϕ*3 × 5 mm	PDLGA *ϕ*3 × 5 mm	48 w	2nd
7	uHA(40) *ϕ*4 × 7 mm	β-TCP(40) *ϕ*4 × 7 mm	uHA(40) *ϕ*3 × 5 mm	defect *ϕ*3 × 5 mm	β-TCP(40) *ϕ*3 × 5 mm	–	6 w	3rd
8							9 w	3rd

Abbreviation: uHA is unsintered hydroxyapatite, β-TCP is β-tricalcium phosphate, PDLGA is poly-dl-lactide-*co*-glycolide, PLGA is poly-l-lactide-*co*-glycolide.

**Table 3:** Anesthesia condition. For anesthesia by intramuscular injection, the anesthetic solution was prepared by mixing Ketalar and Selactar 2% and adjusting the concentration with saline so that the final concentrations were 50 mg/mL of Ketamine and 20 mg/mL of Xylazine and then injected intramuscularly into the rabbit’s lower back. After anesthesia, the rabbit was put to sleep, and CT measurements were performed. The gas anesthesia was initially adjusted to 0.5% Isoflurane and then increased to 1.25% while monitoring the rabbit’s condition, and PET measurements were performed.

Ketamine Hydrochloride	Ketalar for Intravenous Injection (Ketamine: 50 mg/mL)
Xylazine	Selactar 2% injection (Xylazine: 20 mg/mL)
administration	0.5 mL/kg
gaseous anesthetic	Isoflurane inhalant anesthetics Isoflurane: 0.5% → 1.25% O_2_ (0.5 mL/min)/air (0.5 L/min)

## Data Availability

The original contributions presented in this study are included in the article/Supplementary Materials. Further inquiries can be directed to the corresponding author.

## Supplementary Materials

The following supporting information can be downloaded at: https://www.mdpi.com/article/10.3390/ijms27062549/s1.
