# Supplementary material for: Time-Course Evaluation of the In Vivo Resorption Process of Calcium Phosphates/Poly(lactide-co-glycolide) Composites Using Radiological Imaging and Histology
Source: Int J Mol Sci. 2026 Mar 10;27(6):2549. doi: 10.3390/ijms27062549 (PMC13026516; doi:10.3390/ijms27062549)
Supplement: Supplementary file 1 [file ijms-27-02549-s001.zip › ijms-4152086-supplementary.pdf]

Supplementary Figure S1

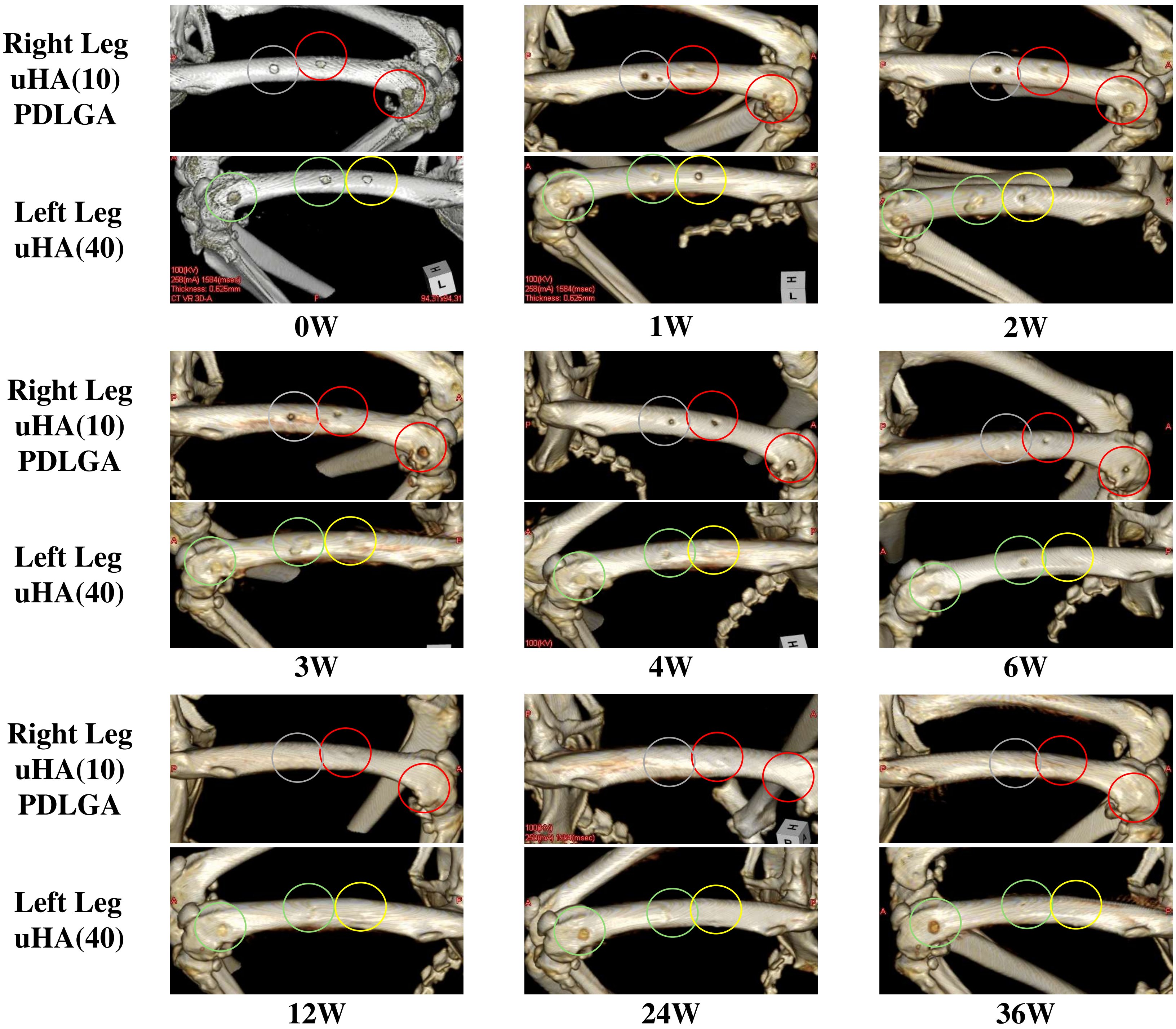

Supplementary Figure S1. Changes in appearance of CT volume rendering images of materials implanted in Rabbit No. 6. Circles indicate the sites where the materials were implanted (blue: β-TCP(40), red: uHA(40), yellow: none (defect)).

Supplementary Figure S2

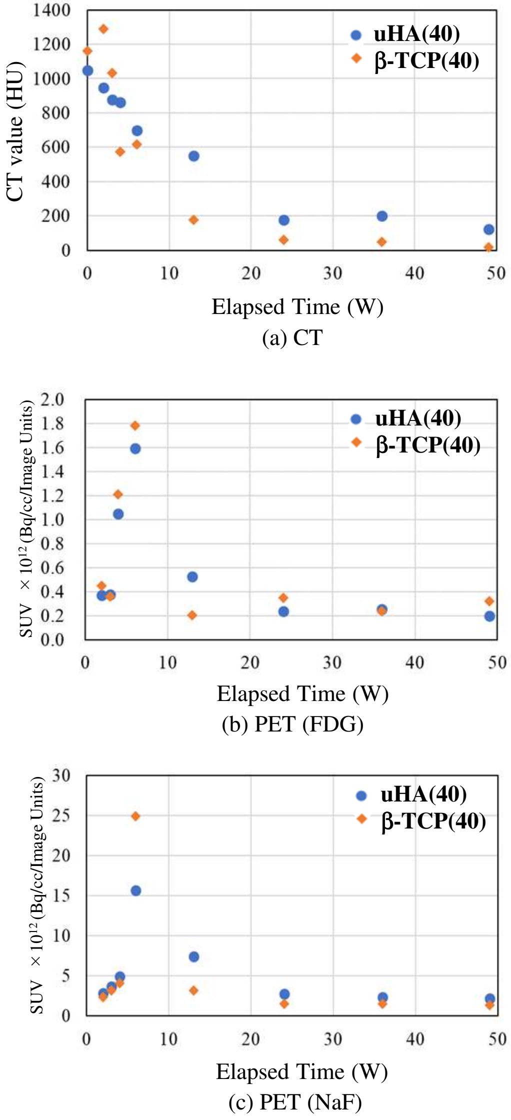

Supplementary Figure 2S-1

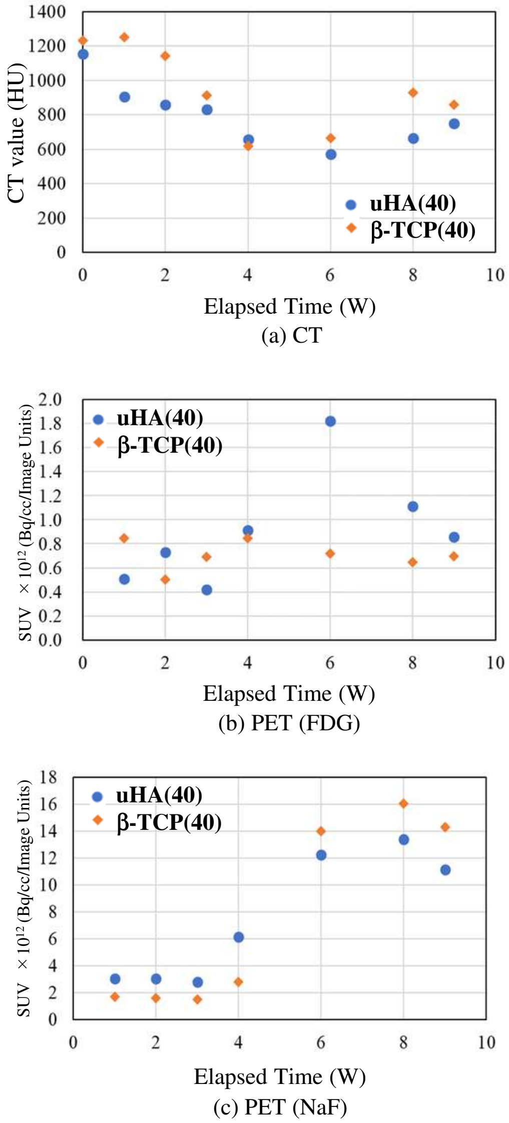

Supplementary Figure 2S-2

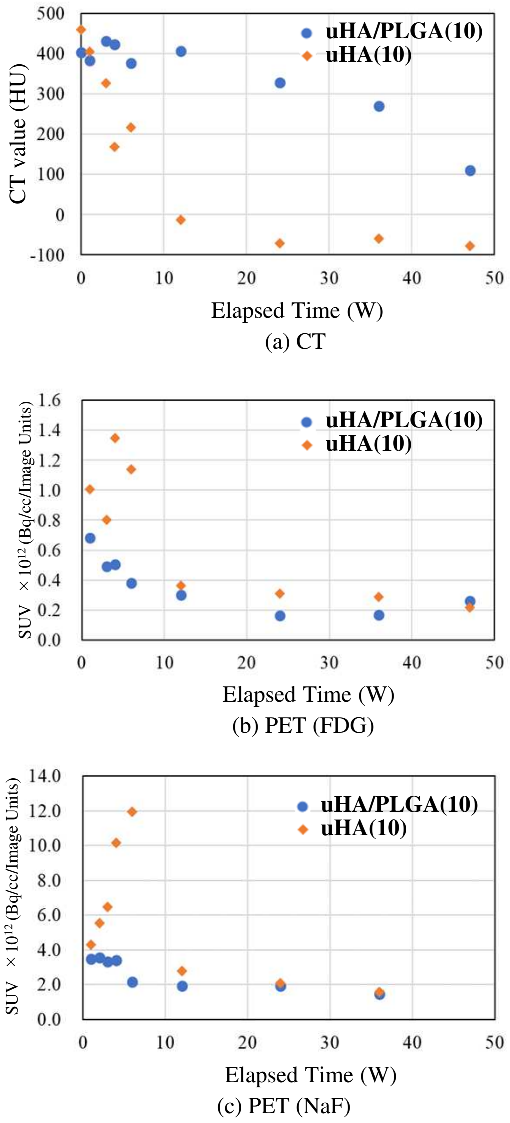

Supplementary Figure 2S-3

Supplementary Figure S2. Quantitative results of CT and PET in the bone marrow region at the femoral metaphysis.

Supplementary Figure S3

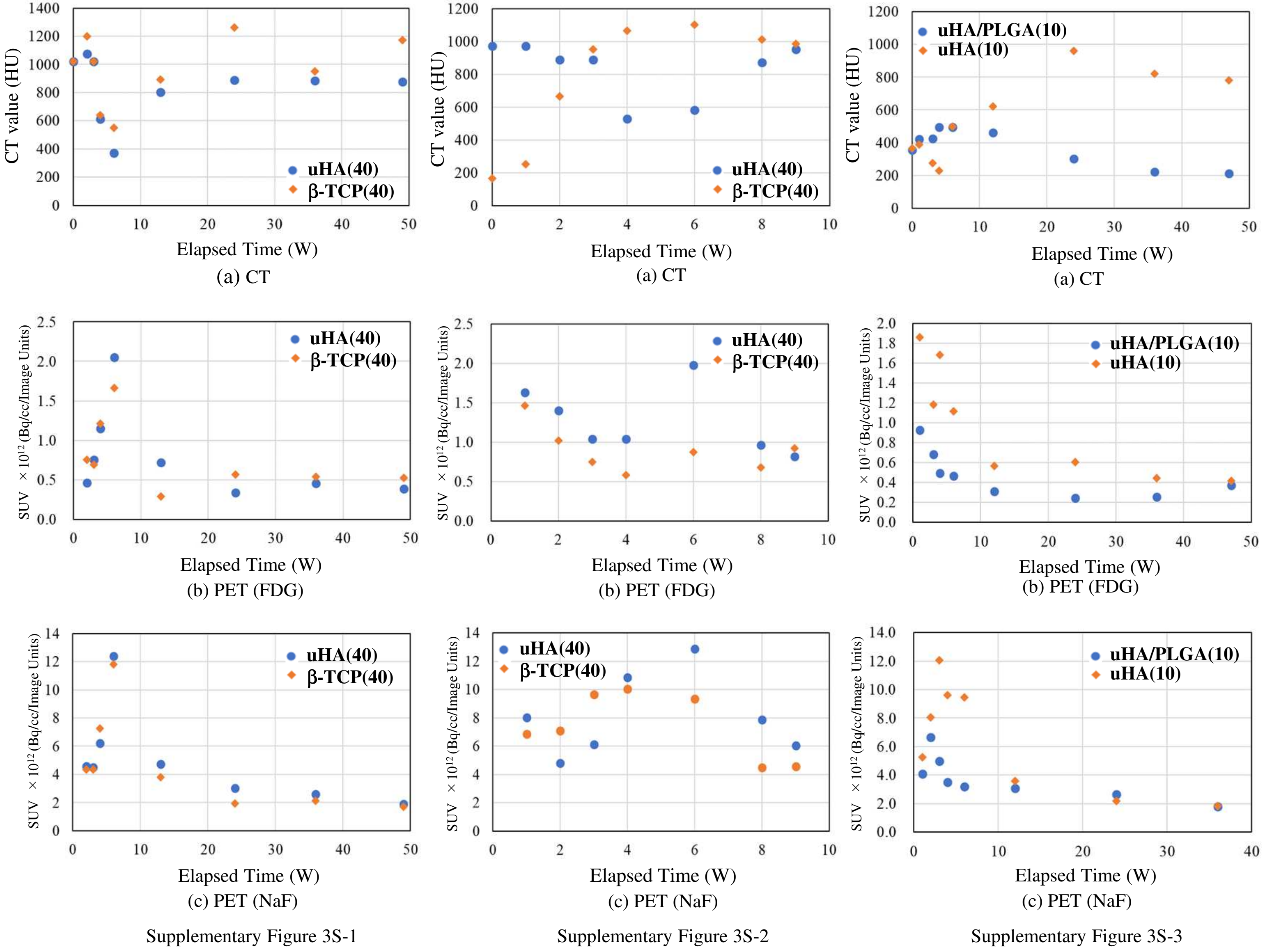

Supplementary Figure S3. Quantitative results of CT and PET in the cortical bone region at the femoral metaphysis.

Supplementary Figure S4

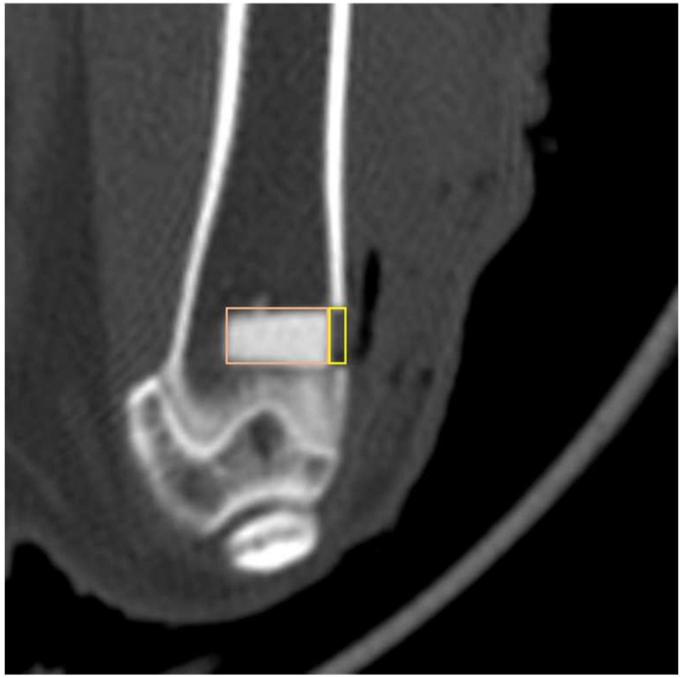

Supplementary Figure S4. A representative image of ROI at the femoral metaphysis. The orange-colored box and the yellow box indicate ROIs set in the bone marrow region and the cortical bone region, respectively.

Supplementary Figure S5

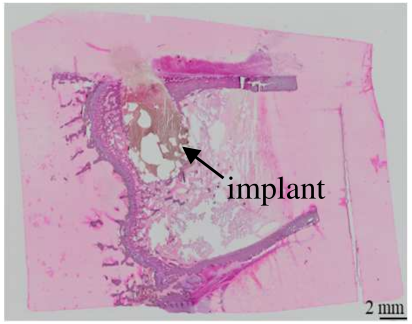

(a)

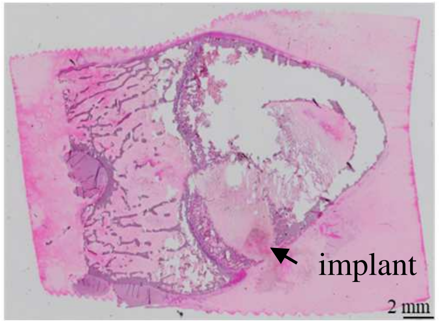

(b)

Supplementary Figure S5. Representative histological images of metaphyseal region in Rabbit-No. 1, sacrificed 1 week after implantation surgery. (a) HE image around the implant (uHA(40)) in the left metaphysis. (b)) HE image around the implant (β-TCP(40)) in the right metaphysis.

Supplementary Figure S6

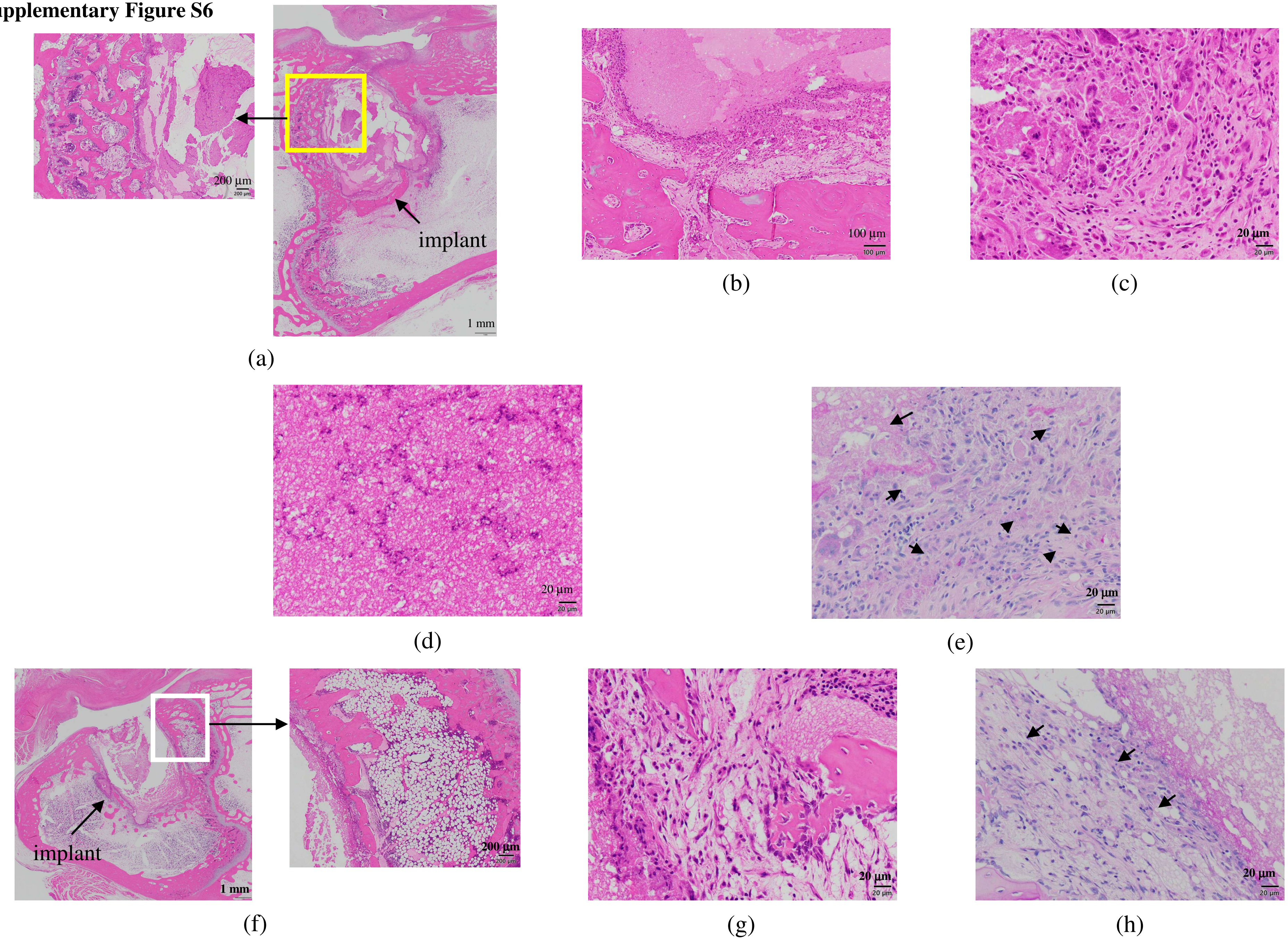

Supplementary Figure S6. Representative histological images of metaphyseal region in Rabbit-No. 4, sacrificed 4 weeks after implantation surgery. (a) HE image around the implant (uHA(40)) in the left metaphysis. (b) HE image in the implant (uHA(40)) in the left metaphysis (×100). (c) HE image in the implant (uHA(40)) in the left metaphysis (×400). (d) HE image in the implant (uHA(40)) in the left metaphysis (×400). (e) PAS image in the implant (uHA(40)) in the left metaphysis (×400). (f) HE image around the implant (β-TCP) in the right metaphysis. (g) HE image in the implant (β-TCP(40)) in the right metaphysis (×400). (h) PAS image in the implant (β-TCP(40)) in the right metaphysis (×400).

Supplementary Figure S7

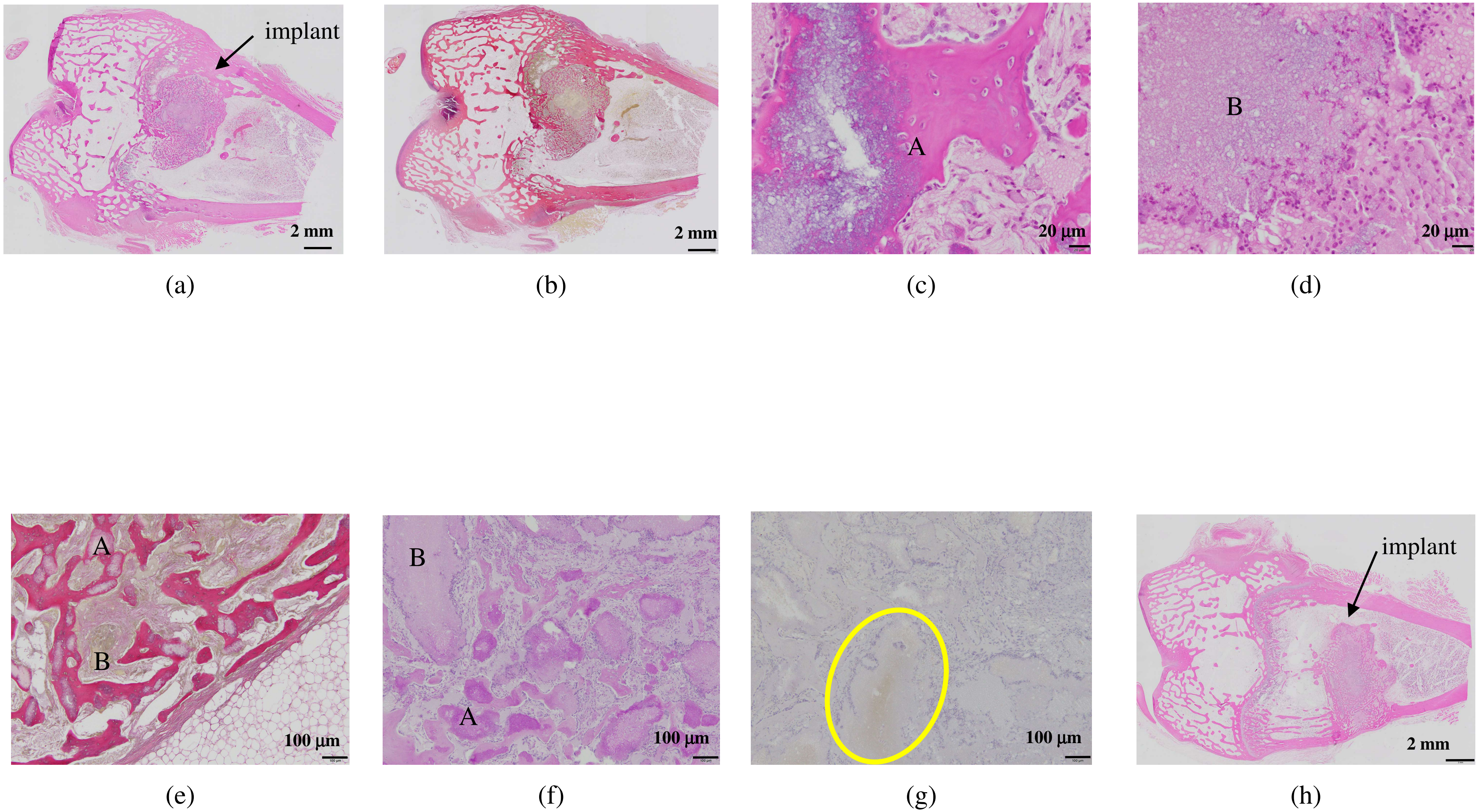

Supplementary Figure S7

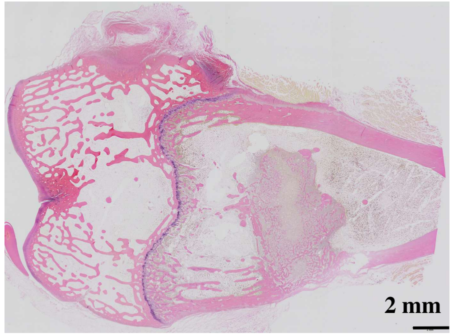

(i)

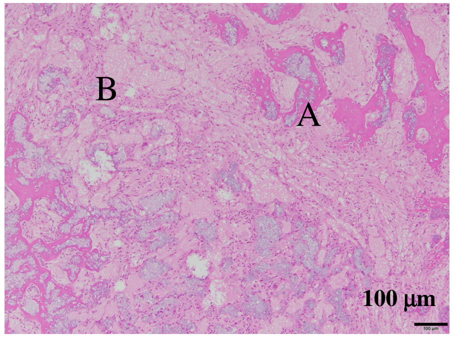

(j)

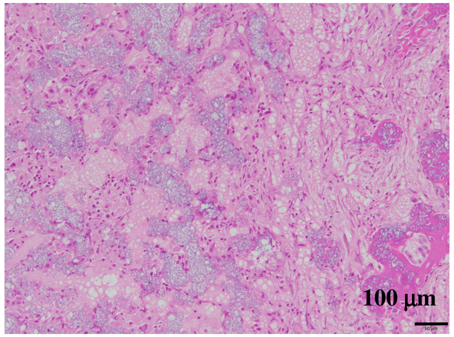

(k)

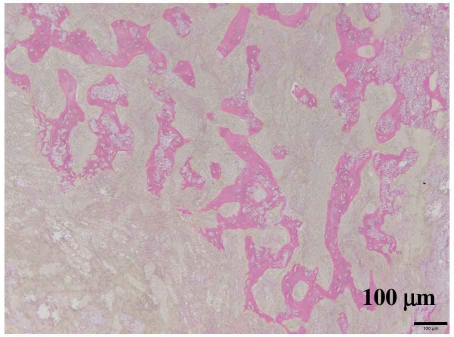

(l)

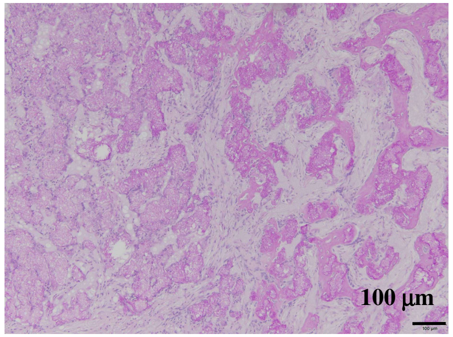

(m)

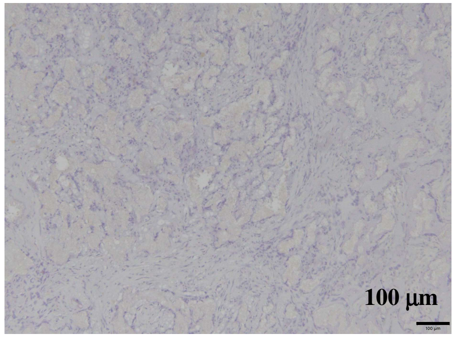

(n)

Supplementary Figure S7. Representative histological images of metaphyseal region in Rabbit-No. 7, sacrificed 6 weeks after implantation surgery. (a) HE image around implant (uHA(40)) in the left metaphysis. (b) EVG image around implant (uHA(40)) in the left metaphysis. (c) HE image in implant (uHA(40)) in the left metaphysis (×400). (d) HE image in the implant (uHA(40)) in the left metaphysis (×400). (e) EVG image in the implant (uHA(40)) in the left metaphysis (×100). (f) PAS image in the implant (uHA(40)) in the left metaphysis (×100). (g) OC image in implant (uHA(40)) in the left metaphysis (×100). (h) HE image around implant (β-TCP(40)) in the right metaphysis. (i) EVG image around implant (β-TCP(40)) in the right metaphysis. (j) HE image in implant (β-TCP(40)) in the right metaphysis (×100). (k) HE image in the implant (β-TCP(40)) in the right metaphysis. (l) EVG image in the implant (β-TCP(40)) in the right metaphysis (×100). (m) PAS image in the implant (β-TCP(40)) in the right metaphysis (×100). (n) OC image in the implant (β-TCP(40)) in the right metaphysis (×100).

Supplementary Figure S8

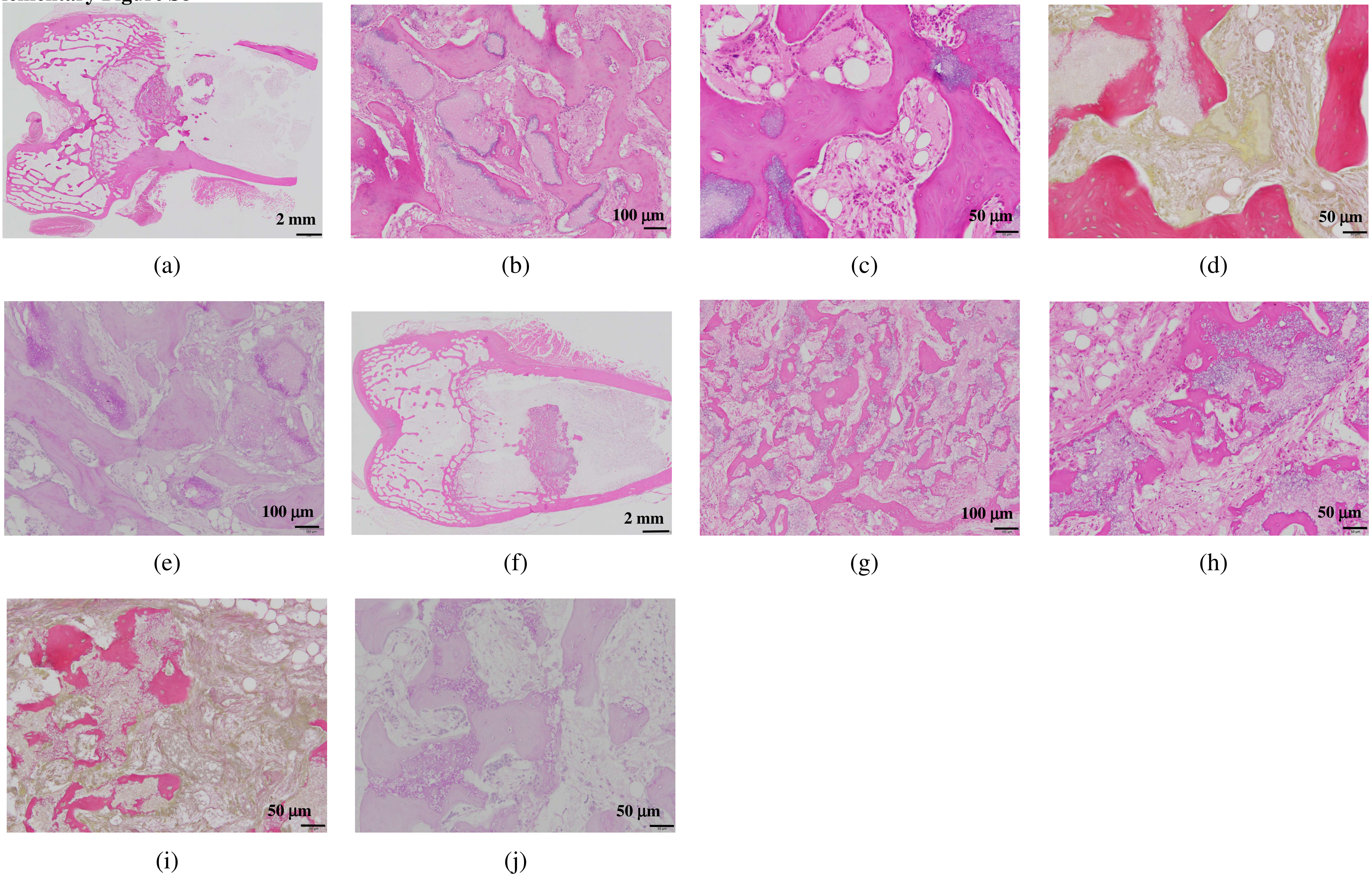

Supplementary Figure S8. Representative histological images of metaphyseal region in Rabbit-No. 8, sacrificed 9 weeks after implantation surgery. (a) HE image around the implant (uHA(40)) in the left metaphysis. (b) HE image in the implant (uHA(40) in the left metaphysis (×100). (c) HE image in the implant (uHA(40)) in the left metaphysis(×200). (d) EVG image in the implant (uHA(40)) in the left metaphysis(×200). (e) PAS image in the implant (uHA(40)) in the left metaphysis(×200). (f) HE image around the implant (β-TCP(40)) in the right metaphysis. (g) HE image around the implant (β-TCP(40)) in the right metaphysis (×100). (h) HE image in the implant (β-TCP(40)) in the right metaphysis (×200). (i) EVG image in the implant (β-TCP(40)) in the right metaphysis (×200). (j) PAS image in the implant (β-TCP(40)) in the right metaphysis (×200).

Supplementary Figure S9

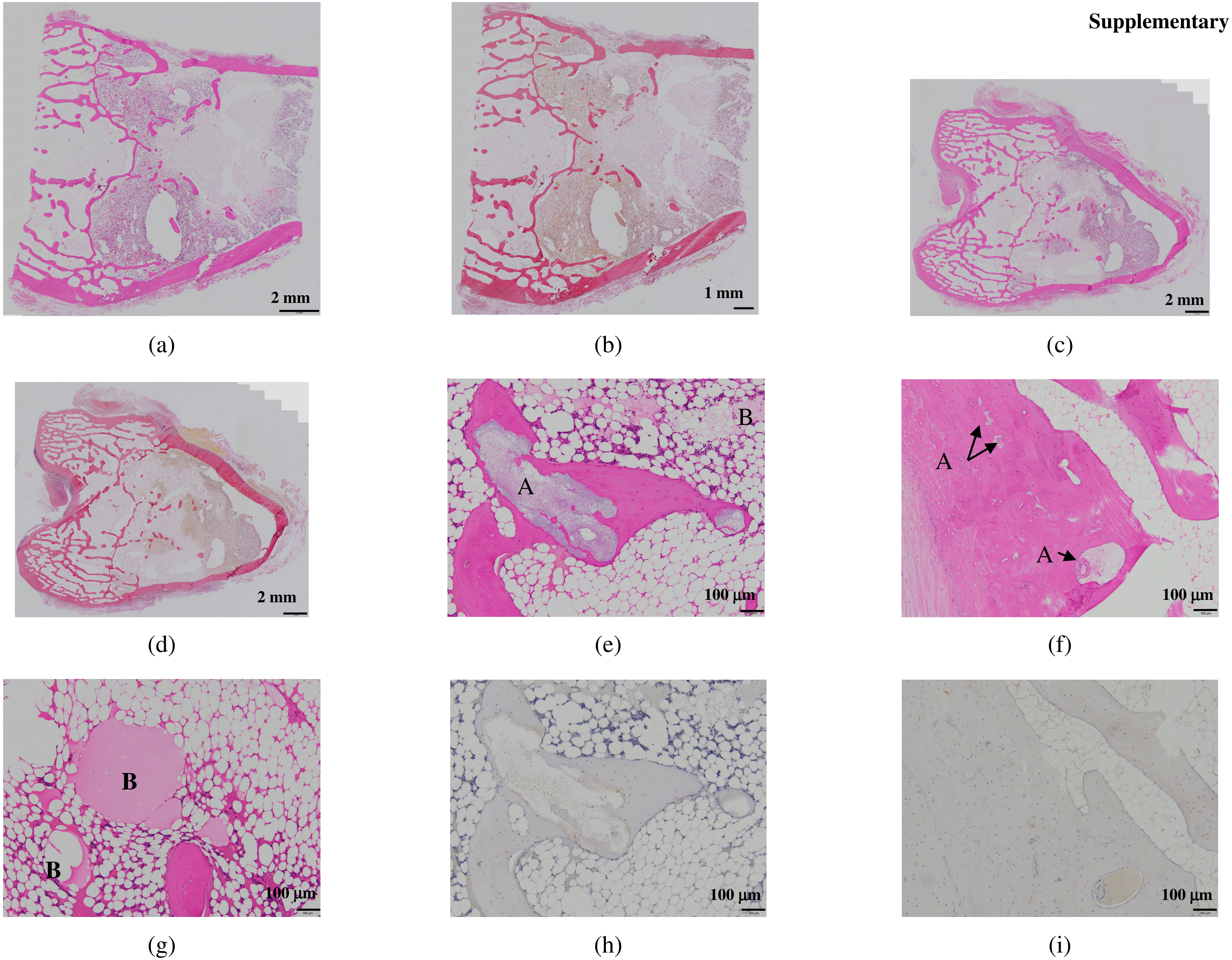

Supplementary Figure S9. Representative histological images of metaphyseal region in Rabbit-No. 3, sacrificed 48 weeks after implantation surgery. (a) HE image around implant (uHA(40)) in the left metaphysis. (b) EVG image around implant (uHA(40)) in the left metaphysis. (c) HE image around implant (β-TCP(40)) in the right metaphysis. (d) EVG image around implant (β-TCP(40)) in the right metaphysis. (e) HE image in implant (uHA(40)) in the left metaphysis (×100). (f) HE image in implant (β-TCP(40)) n the right metaphysis (×100). (g) HE image in implant (β-TCP(40)) in the right metaphysis (×100). (h) OC image in implant (uHA(40)) in the left metaphysis (×100). (i) OC image in implant (β-TCP(40)) in the right metaphysis (×100).

Supplementary Figure S10

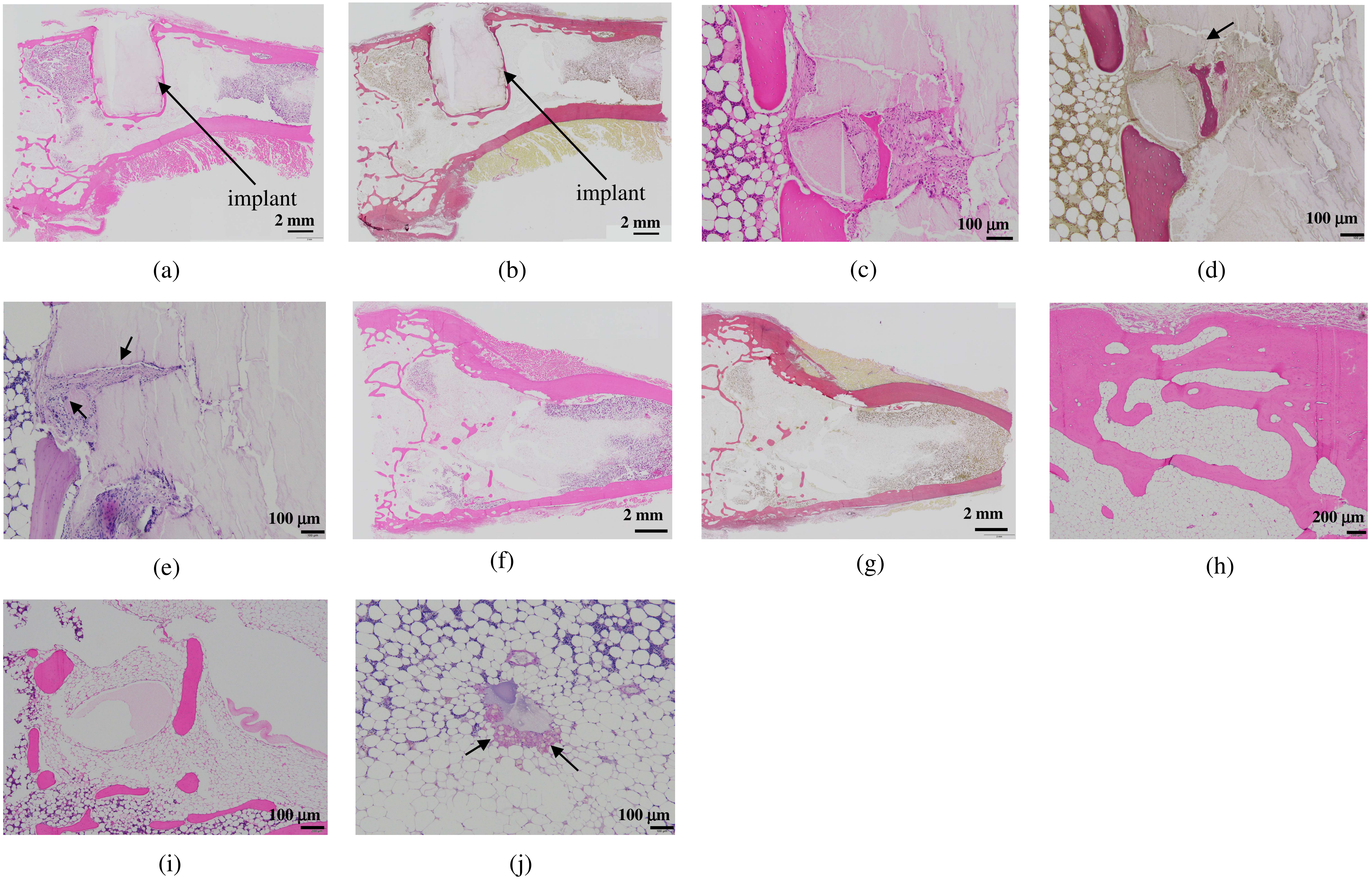

Supplementary Figure S10. Representative histological images of metaphyseal region in Rabbit-No. 6, sacrificed 48 weeks after implantation surgery. (a) HE image around the implant (uHA/PLGA(10)) in the left metaphysis. (b) EVG image around the implant (uHA/PLGA(10)) in the left metaphysis. (c) HE image in the implant (uHA/PLGA(10)) in the left metaphysis (×100). (d) EVG image in the implant (uHA/PLGA(10)) in the left metaphysis (×100). (e) PAS image around the implant (uHA/PLGA(10)) in the left metaphysis (×100). (f) HE image around the implant (uHA (10)) in the right metaphysis. (g) EVG image around the implant (uHA (10)) in the right metaphysis. (h) HE image in the implant (uHA (10)) in the right metaphysis (x40). (i) HE image in the implant (uHA (10)) in the right metaphysis (×40). (j) PAS image in the implant (uHA (10)) in the right metaphysis (×100).

Supplementary Figure S11

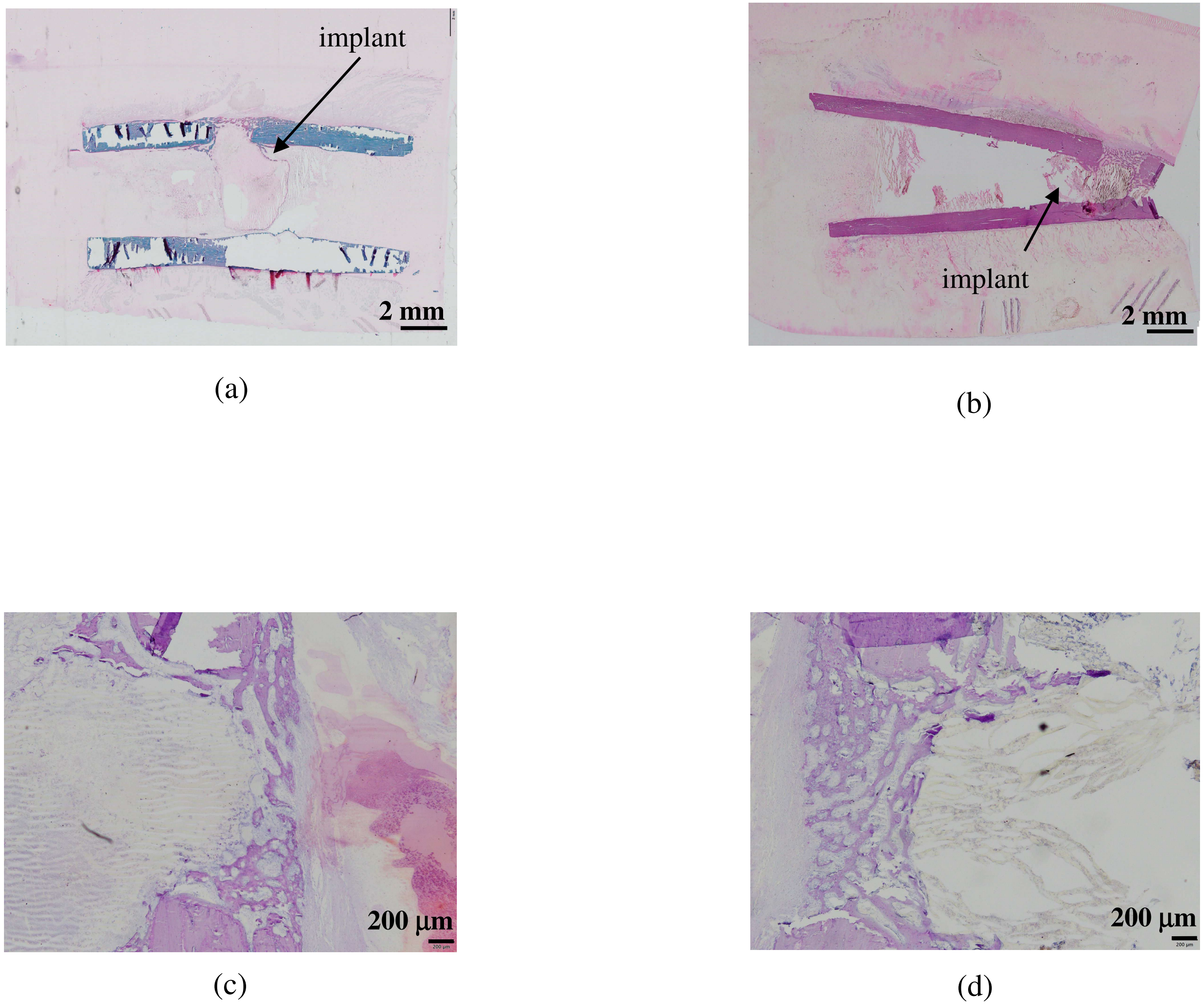

Supplementary Figure S11. Representative histological images of diaphyseal region in Rabbit-No. 1, sacrificed 1 week after implantation surgery. (a) HE image around the implant (uHA (40)) in the left diaphysis. (b) HE image around the implant (β-TCP (40)) in the left diaphysis. (c) PAS image around the implant (uHA (40)) in the left diaphysis. . (d) PAS image around the implant (β-TCP (40)) in the left diaphysis (x40).

Supplementary Figure S12

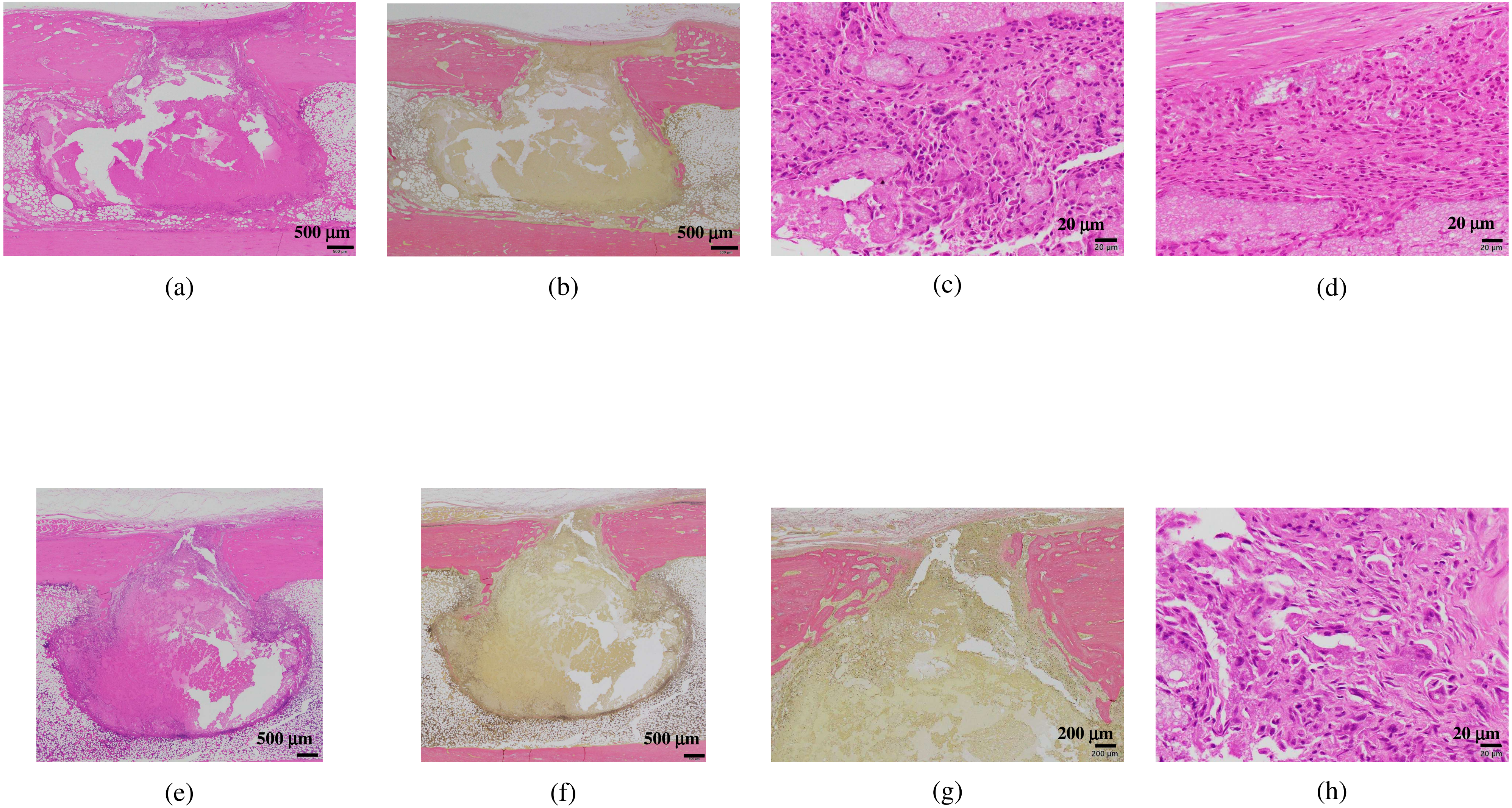

Supplementary Figure S12. Representative histological images of diaphyseal region in Rabbit-No. 4, sacrificed 4 weeks after implantation surgery. (a) HE image around the implant (uHA (40)) in the left diaphysis. (b) EVG image around the implant (uHA (40)) in the left diaphysis. (c) HE image in the implant (uHA (40)) in the left diaphysis (×400). (d) HE image in the implant (uHA (40)) in the left diaphysis. (h) HE image in the implant (β-TCP (40)) in the left diaphysis. (e) HE image around the implant (β-TCP(40)) in the left diaphysis. (f) EVG image around the implant (β-TCP(40)) in the left diaphysis. (g) EVG image around the implant (β-TCP(40)) in the left diaphysis (×40). (h) HE image in the implant (uHA (40)) in the right diaphysis (×400).

Supplementary Figure S13

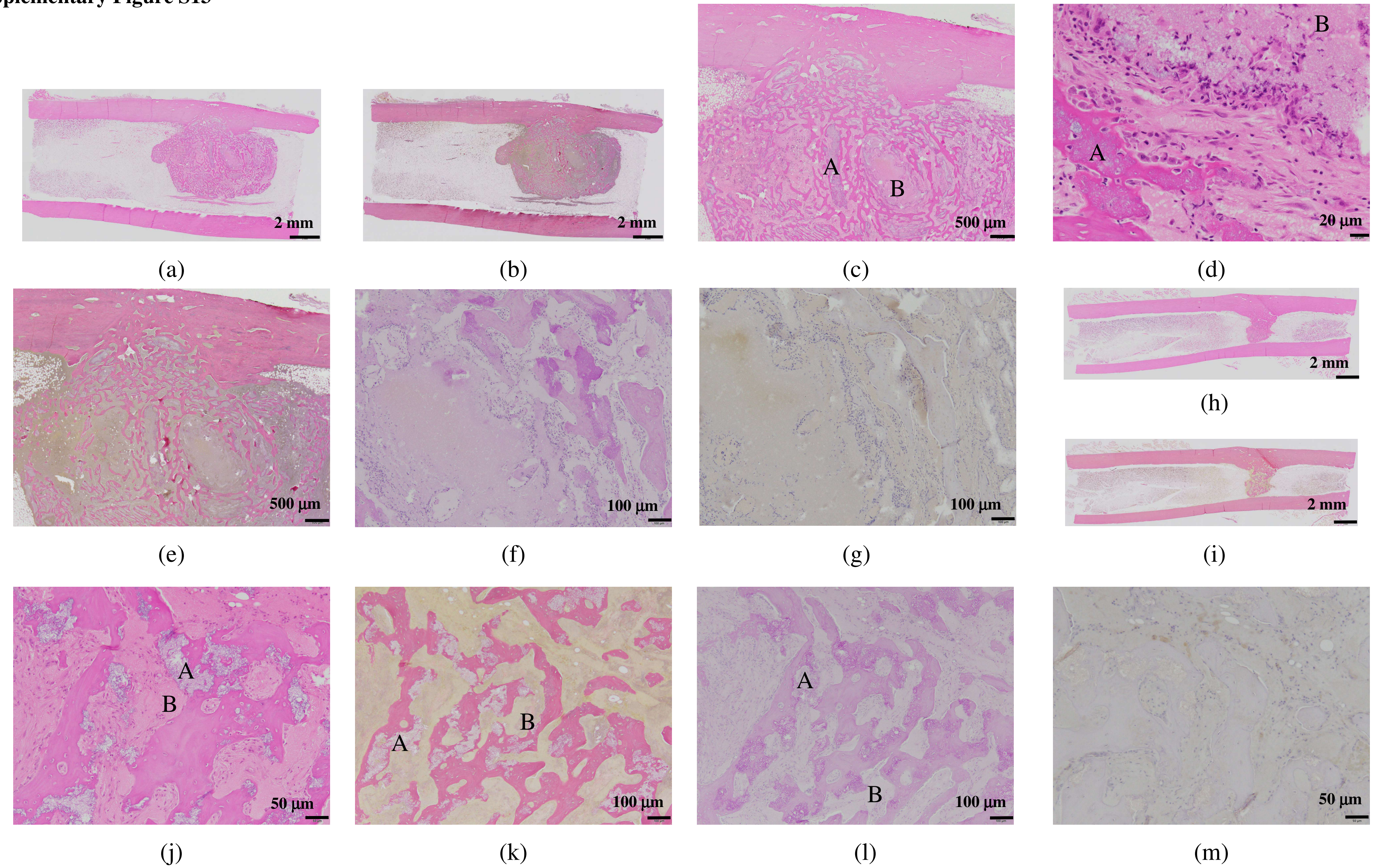

Supplementary Figure S13. Representative histological images of diaphyseal region in Rabbit-No. 7, sacrificed 6 weeks after implantation surgery. (a) HE image around the implant (uHA(40)) in the left diaphysis. (b) EVG image around the implant (uHA(40)) in the left diaphysis. (c) HE image around the implant (uHA(40)) in the left diaphysis (×20). (d) HE image in the implant (uHA(40)) in the left diaphysis (×400). (e) EVG image around the implant (uHA(40)) in the left diaphysis (×20). (f) PAS image in the implant (uHA(40)) in the left diaphysis (×100). (g) OC image in the implant (uHA(40)) in the left diaphysis (×100). (h) HE image around the implant (β-TCP(40)) in the right diaphysis. (i) EVG image around the implant (β-TCP(40)) in the right diaphysis. (j) HE image in the implant (β-TCP(40)) in the right diaphysis (×200). (k) EVG image in the implant (β-TCP(40)) in the right diaphysis (×100). (l) PAS image in the implant (β-TCP(40)) in the right diaphysis (×100). (m) OC image in the implant (β-TCP(40)) in the left diaphysis (×100).

Supplementary Figure S14

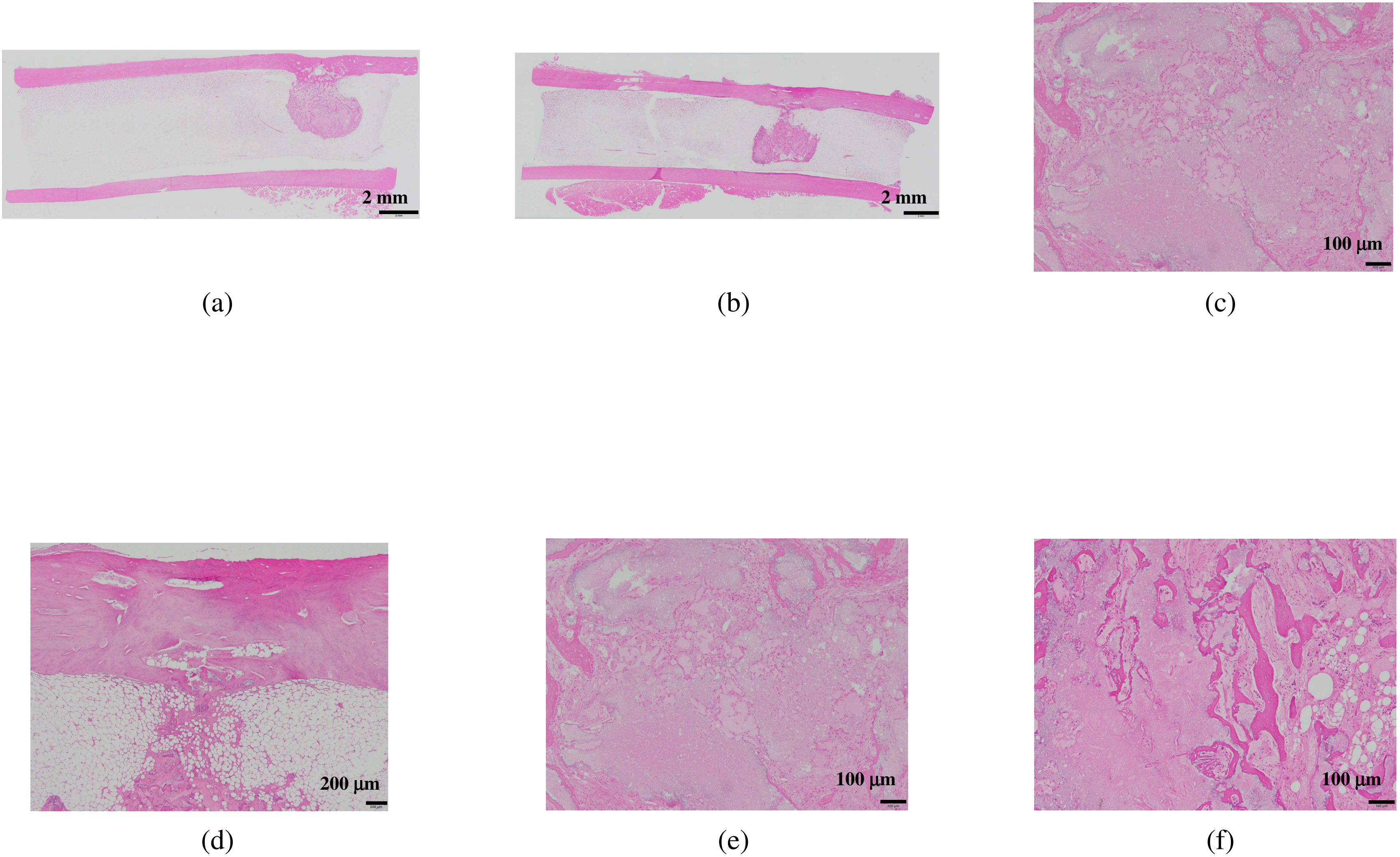

Supplementary Figure S14. Representative histological images of diaphyseal region in Rabbit-No. 8, sacrificed 9 weeks after implantation surgery. (a) HE image around the implant (uHA(40)) in the left diaphysis. (b) HE image around the implant (β-TCP(40)) in the right diaphysis. (c) HE image in the implant (uHA(40)) in the left diaphysis (×40). (d) HE image in the implant (β-TCP(40)) in the right diaphysis (×40). (e) HE image in the implant (uHA(40)) in the left diaphysis (×100). (f) HE image in the implant (β-TCP(40)) in the right diaphysis (×100).

Supplementary Figure S15

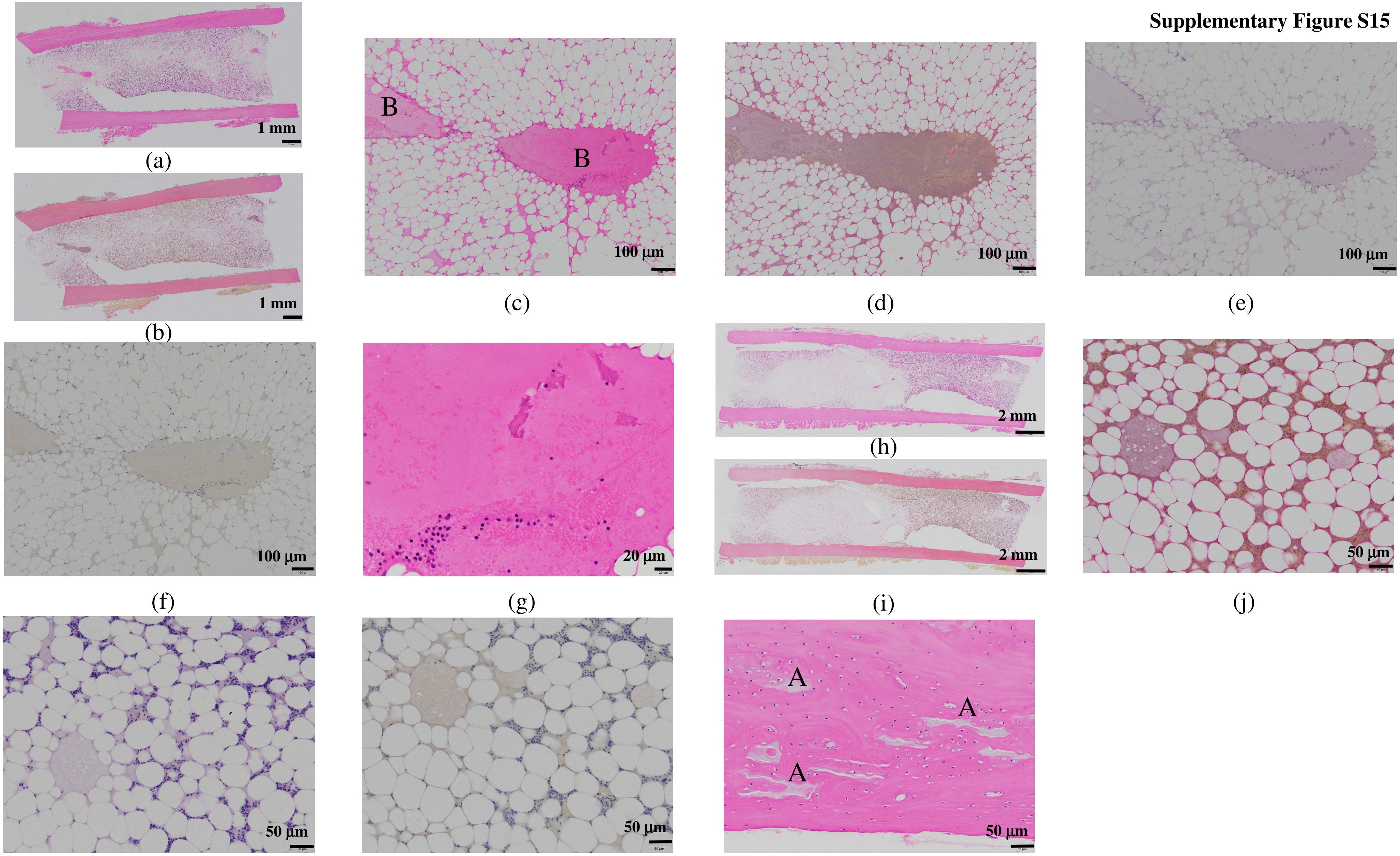

(a) (b) (c) (d) (e) (f) (g) (h) (i) (j) (k) (l) (m)

Supplementary Figure S15. Representative histological images of diaphyseal region in Rabbit-No. 3, sacrificed 48 weeks after implantation surgery. (a) HE image around the implant (uHA(40)) in the left diaphysis. (b) EVG image around the implant (uHA(40)) in the left diaphysis. (c) HE image in the implant (uHA(40)) in the left diaphysis (×100). (d) EVG image in the implant (uHA(40)) in the left diaphysis (×100). (e) PAS image in the implant (uHA(40)) in the left diaphysis (×100). (f) OC image in the implant (uHA(40)) in the left diaphysis (×100). (g) HE image in the implant (uHA(40)) in the left diaphysis (×400). (h) HE image around the implant (β-TCP(40)) in the right diaphysis. (i) EVG image around the implant (β-TCP(40)) in the right diaphysis. (j) EVG image in the implant (β-TCP(40)) in the right diaphysis (×200). (k) PAS image in the implant (β-TCP(40)) in the right diaphysis (×200). (l) OC image in the implant (β-TCP(40)) in the right diaphysis (×200). (m) HE image in the implant (β-TCP(40)) in the right diaphysis.

Supplementary Figure S16

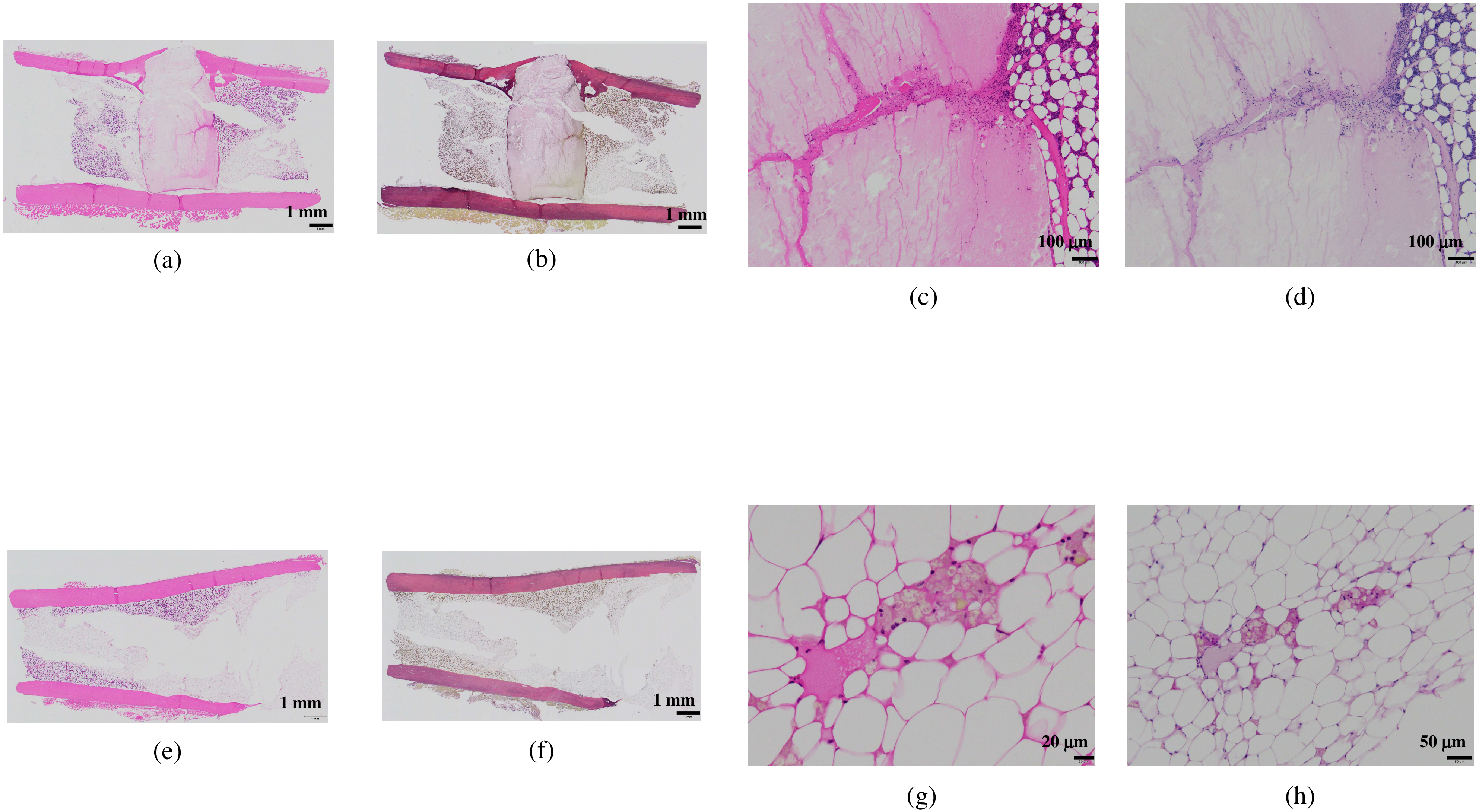

Supplementary Figure S16. Representative histological images of diaphyseal region in Rabbit-No. 6, sacrificed 48 weeks after implantation surgery.(a) HE image around the implant (uHA/PLGA(10)) in the left diaphysis. (b) EVG image around the implant (uHA/PLGA(10)) in the left diaphysis. (c) HE image in the implant (uHA/PLGA(10)) in the left diaphysis (×100). (d) PAS image in the implant (uHA/PLGA(10)) in the left diaphysis (×100). (e) HE image around the implant (uHA(10) in the right diaphysis. (f) EVG image around the implant (uHA(10)) in the right diaphysis. (g) HE image in the implant (uHA (10)) in the right diaphysis (×400). (h) PAS image in the implant (uHA (10)) in the right diaphysis (×200).

Supplementary Figure S17

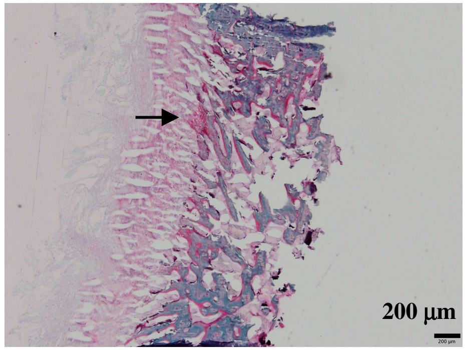

(a)

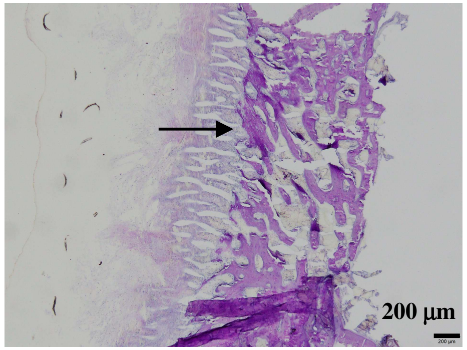

(b)

Supplementary Figure S17. Representative histological images of bone defect area of diaphysis in Rabbit-No. 1, sacrificed 1 week after implantation surgery. (a) VG image around bone defect in the left diaphysis (×40). (b) PAS image around bone defect in the left diaphysis.

Supplementary Figure S18

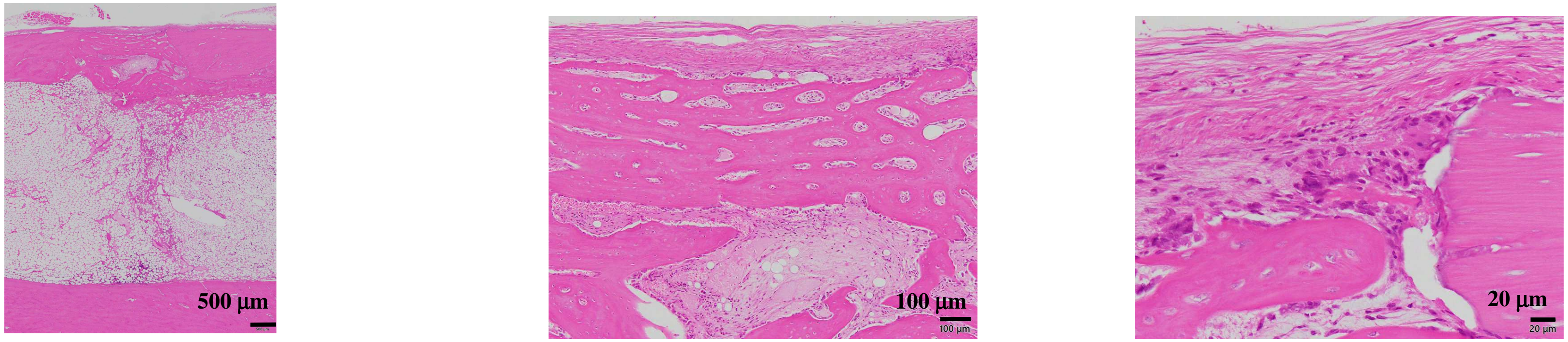

(a) (b) (c)

Supplementary Figure S18. Representative histological images of bone defect area of diaphysis in Rabbit-No. 4, sacrificed 4 weeks after implantation surgery. (a) HE image around the born defect in the left diaphysis. (b) HE image in the born defect in the left diaphysis (×100). (c) HE image in the born defect in the left diaphysis (×100).

Supplementary Figure S19

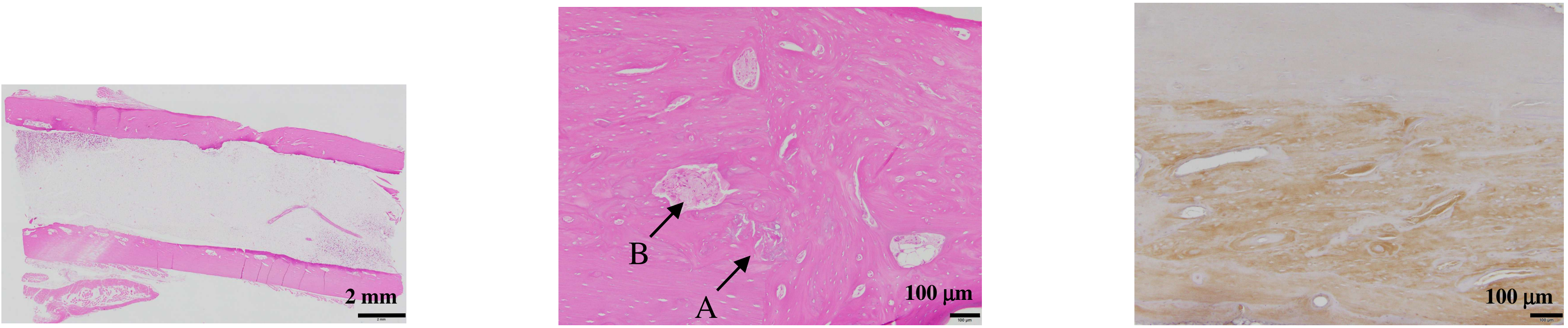

(a) (b) (c):

Supplementary Figure S19. Representative histological images of bone defect area of diaphysis in Rabbit-No. 7, sacrificed 6 weeks after implantation surgery. (a) HE image around the born defect in the left diaphysis. (b) HE image in the born defect in the left diaphysis (×100). (c) OC image in the born defect in the left diaphysis (×100).

Supplementary Figure S20

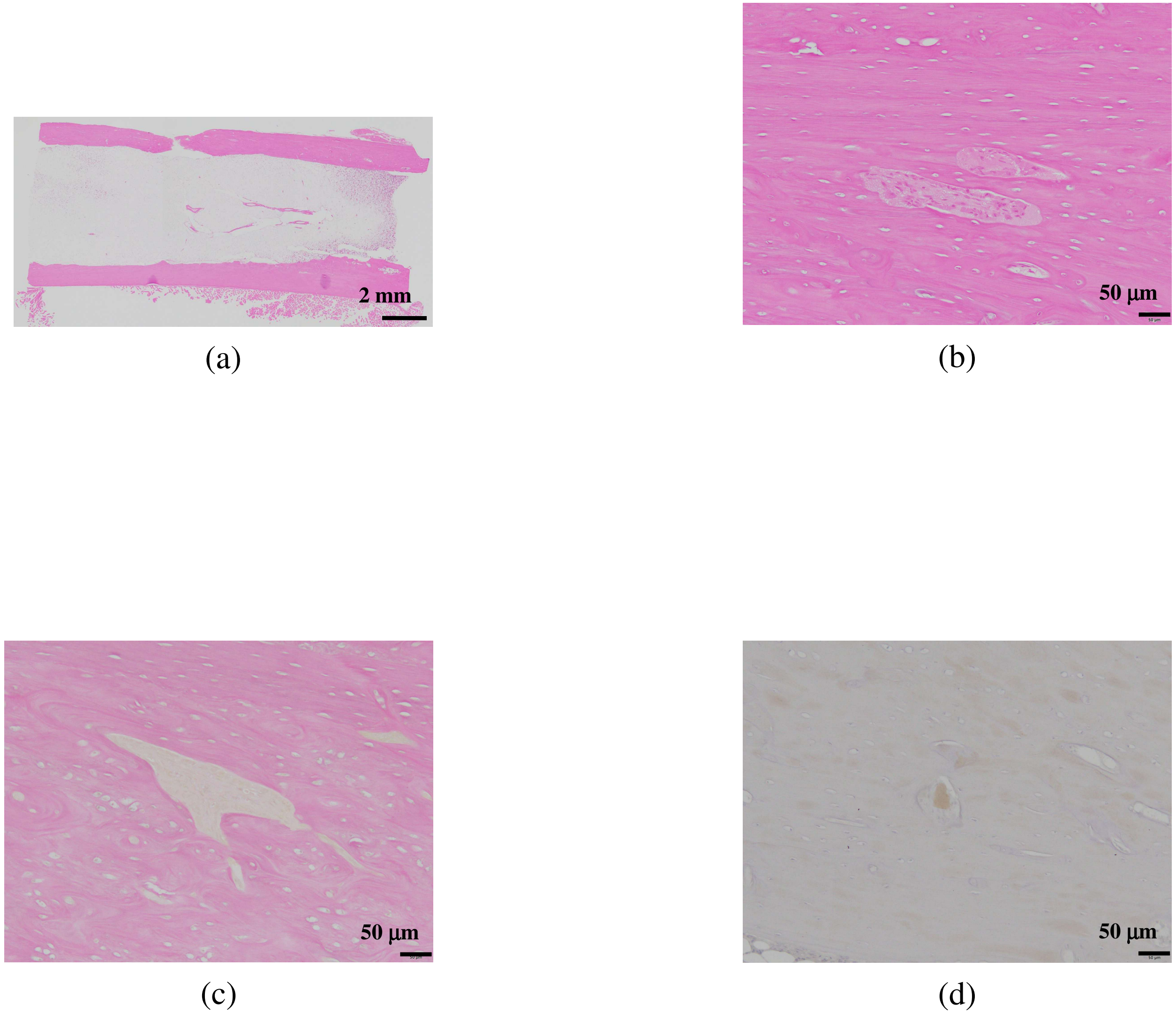

Supplementary Figure S20. Representative histological images of bone defect area of diaphysis in Rabbit-No. 8, sacrificed 9 weeks after implantation surgery. (a) HE image around the born defect in the left diaphysis. (b) HE image in the born defect in the left diaphysis (×200). (c) EVG image in the born defect in the left diaphysis (×200). (d) OC image in the born defect in the left diaphysis (×200).

Supplementary Figure S21

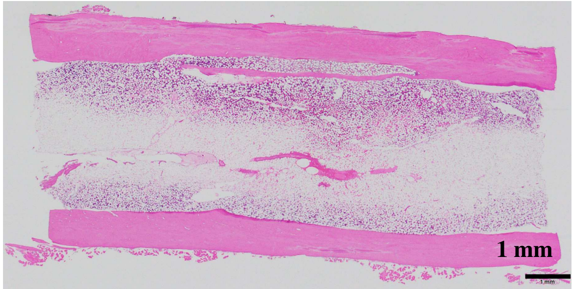

Supplementary Figure S21. Representative histological images of bone defect area of diaphysis in Rabbit-No. 3, sacrificed 48 weeks after implantation surgery stained by HE.

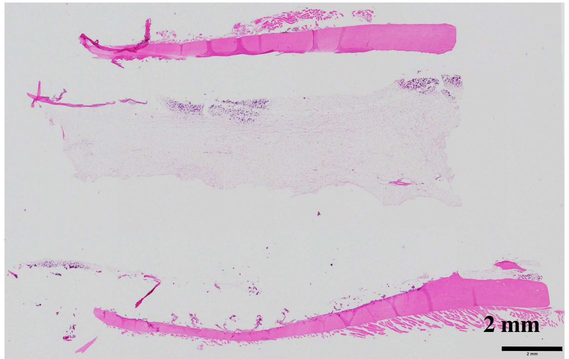

Supplementary Figure S22. Representative histological images of bone defect area of diaphysis in Rabbit-No. 6, sacrificed 48 weeks after implantation surgery stained by HE.

Supplementary Figure S23

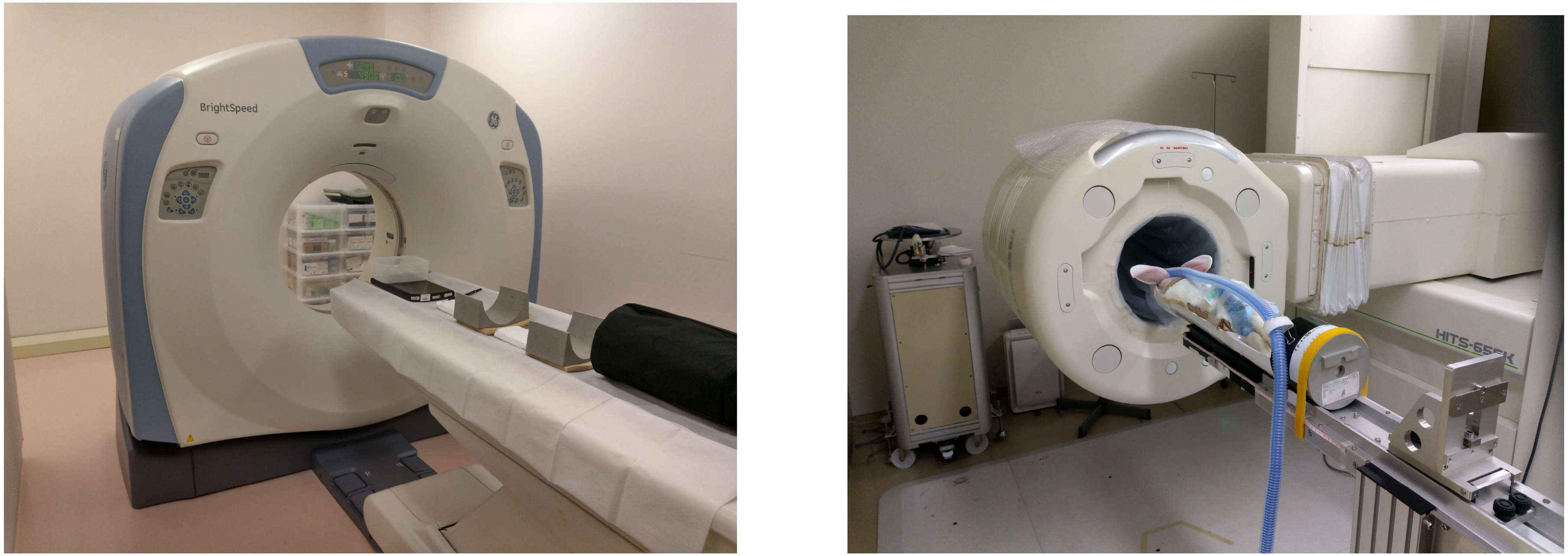

(a) (b)

Supplementary Figure S23. CT equipment and PET equipment and bed for rabbits. (a) X-ray computed tomography made by GE (Bright Speed 16ch). (b) Custom made PET.

Supplementary Figure S24

Weight measurement

↓

Intravenous injection

Ketamine hydrochloride and Xylazine

↓

CT measurement

↓

Move to PET preparation room

↓

Blood sampling （only in the case of FDG）

↓

Intravenous injection (FDG or NaF)

↓

15 min. after injection of FDG or NaF,
start Isoflurane inhalant anesthetics

↓

Setting the rabbit on the PET device
Move the rabbit to PET scanner under Isoflurane inhalant anesthetics

↓

Positioning the rabbit

↓

PET measurement

↓

49 min.～59 min. after injection of FDG

109 min.～119 min. after injection of NaF

Supplementary Figure S24. Experimental flow of PET-CT.

Supplementary Table S1

Supplementary Table S1 Detailed information of rabbits used in the experiment.

Rabbit No.	Initial Weight (kg)	Type	Age (weeks)	Sex	Breeding Site	Feed	Provider
1	2.8	Kbs: Jw	13	Male	Laboratory Animal-Breeding Facilities in Hamamatsu University School of medicine	5L95 Product of LabDiet	Oriental Yeast Co., Ltd.
2	2.9						
3	2.8						
4	2.6						
6	2.6						
7	2.9						
8	2.8						
